# Dietary supplement for mood symptoms in early postpartum: a double-blind randomized placebo controlled trial

**DOI:** 10.1016/j.eclinm.2024.102593

**Published:** 2024-04-10

**Authors:** Jeffrey H. Meyer, ZhaoHui Wang, Apitharani Santhirakumar, Yekta Dowlati, Natalia Docteur, Aqsa Shoaib, Jareeat Purnava, Yanqi Wang, Wei Wang, Sheng Chen, Muhammad I. Husain, Rashmi de Silva Wijeyeratne, Heba Reeyaz, Catalina Baena-Tan, Yuko Koshimori, Zahra Nasser, Valery Sit

**Affiliations:** a Brain Health Imaging Centre, Azrieli Centre for Neuro-Radiochemistry, Campbell Family Mental Health Research Institute, Centre for Addiction and Mental Health (CAMH), 250 College Street, Toronto, M5T 1R8, Canada; b Department of Psychiatry, University of Toronto, 250 College Street, 8th Floor, Toronto, M5T 1R8, Canada

**Keywords:** Postpartum blues, Postpartum depression, Tryptophan, Tyrosine, Antioxidants

## Abstract

**Background:**

Postpartum blues (PPB) is a frequent syndrome of sad mood, crying spells, anxiety, restlessness, reduced appetite, and irritability, typically peaking day 5 postpartum. When severe, it greatly increases risk for later postpartum depression. This trial compared a dietary supplement to placebo on PPB severity. The supplement was designed to counter downstream effects of elevated monoamine oxidase A level, implicated in causing PPB.

**Methods:**

Participants recruited by advertisement from the Toronto region completed procedures at CAMH, Canada and/or participants’ homes. Oral supplement or identical appearing relatively inert placebo were administered in randomised, double-blind fashion. Supplement was blueberry juice and extract given four times between nighttime day 3 and morning day 5 postpartum; tryptophan 2 g nighttime day 4 postpartum, and tyrosine 10 g morning day 5 postpartum. On day 5, depressed mood induction procedure (MIP) and postpartum blues were assessed. All data is presented (NCT03296956 closed, clinicaltrials.gov).

**Findings:**

Between January 2019 and December 2022, participants took supplement (n = 51) or placebo (n = 52). There was no significant effect on primary outcome MIP on visual analogue scale for depressed mood (mean difference = −0.39 mm, 95% CI: −6.42 to 5.65 mm). Stein Maternity Blues scores, exploratory PPB measure, was lower in the active group (effect size 0.62; median, interquartile range (IQR): active 2.00 (IQR 1, 4); placebo 4.00 (IQR 1.5, 6); regression with general linear model, supplement effect, β coefficient = −1.50 (95%: CI −2.60, −0.40), p = 0.008; effect of CES-D crying category before supplement, p = 0.03–0.00000023). Twenty-six and 40 different adverse events occurred within 25% and 42% of supplement and placebo cases respectively (Chi-Square, p = 0.06).

**Interpretation:**

The primary outcome was negative for effect on depressed mood induction, however the supplement moderately reduced PPB.

**Funding:**

CAMH/Exeltis.

**Research in context**

**Evidence before this study**

To identify studies using dietary supplements to prevent or reduce symptoms of postpartum blues or postpartum depression, Pubmed, Psychinfo and Cochrane databases were searched for any relevant articles from 1995 to March 2020 using key words including postpartum blues, postpartum depression, postnatal depression and prevention. The search was done prior to the start of the study and repeated March 23, 2020; and published as part of a review. There was one negative trial of docosahexaenoic acid with or without arachidonic acid versus placebo on postpartum blues. One trial was positive for lower risk scores for postpartum depression within 8 weeks of giving birth after oral selenium supplementation in a region with frequent selenium deficiency.

**Added value of this study**

The supplement reduced postpartum blues with an effect size of 0.62 but did not have effect on depressed mood induction.

**Implications of all the available evidence**

The dietary supplement is well tolerated and reduces postpartum blues severity. This methodological approach may provide a framework for testing other interventions to target postpartum blues in future research.

## Introduction

Postpartum blues is a common syndrome, occurring in up to 75% of women depending on the threshold applied,^1^ that may include episodes of sad mood, crying spells, anxiety, restlessness, reduced appetite, fatigue and irritability. It typically starts around day 3 postpartum, peaks at day 5 postpartum and then decreases over several days to a week.^1^, ^2^, ^3^ Postpartum blues is an important problem because it may be harshly unpleasant; and when severe may be viewed as prodromal for postpartum depression (PPD) since severe postpartum blues raises later risk for PPD at least four fold.^1^, ^2^, ^3^ PPD is a major depressive episode (MDE) with some symptoms starting within a month of giving birth and a MDE being present within several months,^4^ with some definitions extending this latter criterion to 6 months to a year.^5^ Symptoms of MDE, which last at least two weeks, include depressed mood and/or anhedonia at least most of the day; and total at least five symptoms which may include significant weight change, insomnia or hypersomnia, psychomotor agitation or retardation, fatigue or loss of energy, feelings of worthlessness or inappropriate guilt, diminished ability to think or concentrate, or recurrent thoughts of death.^4^ PPD is the most common complication of childbearing at ∼13% prevalence^6^^,^^7^ and there are ∼140 million births globally per year. Unfortunately, evidence based interventions to prevent postpartum blues suitable for widespread use are lacking. The present study compares a dietary supplement to placebo for preventing postpartum blues.

The dietary supplement tested in the present study is intentioned to create resiliency against downstream effects of elevated monoamine oxidase A (MAO-A) level at day 5 postpartum, when postpartum blues are typically most prominent (see Supplementary introduction). MAO-A is the main metabolic pathway of serotonin, and contributes to the metabolism of other monoamines like norepinephrine and dopamine; and in the process generates hydrogen peroxide. Depletion of these monoamines,^8^, ^9^, ^10^ is associated with high risk of precipitating depressive syndromes. Greater MAO-A level is associated with greater MAO-A activity in brain tissue,^11^ so to counter greater magnitude of the functions of MAO-A during early postpartum, several components were included in the dietary supplement. It is composed of blueberry juice and blueberry antioxidants to counter increased production of hydrogen peroxide, l-tryptophan to replace serotonin lost via greater MAO-A metabolism, and l-tyrosine to replace norepinephrine and dopamine also lost via greater MAO-A metabolism. In rodents, during conditions in which release of monoamines is stimulated, administration of l-tryptophan may increase the release of serotonin and administration of l-tyrosine may increase the release of dopamine and norepinephrine^12^, ^13^, ^14^ (also see Supplementary introduction). The latter two ingredients were given at levels greater than those in diet and were previously demonstrated not to affect total level of tryptophan and tyrosine in breast milk.^15^^,^^16^ This was expected because approximately 99% of tryptophan and tyrosine are found as proteins in breast milk so influencing the 1% free portion has negligible effect on overall levels of these amino acids.^15^^,^^16^ In addition, l-tryptophan may help with sleep initiation^17^ and l-tyrosine may improve cognitive performance during stress.^18^

An open trial of this supplement, completed prior to COVID-19, was associated with a substantively reduced magnitude of depressed mood induction on day 5 postpartum.^19^ The primary aim of the present study is to assess the effect of this dietary supplement on postpartum blues on day 5 postpartum. This involves assessing the effect of depressed mood induction and applying a scale for overall severity of postpartum blues. A secondary aim, since severity of postpartum blues is a strong predictor of latter PPD,^1^^,^^2^ is to assess effect of dietary supplement on progression to depressive symptoms for the subsequent six months. The hypotheses are that the dietary supplement will be associated with a lesser severity of postpartum blues and less progression towards depressive symptoms over the subsequent six months.

## Methods

### Study design

This double blind placebo controlled trial was conducted at the Centre for Addiction and Mental Health (CAMH), Toronto, Canada. Participants and all members of the study team were blinded, except for the CAMH pharmacy and the National Sanitation Foundation (NSF) (Guelph, Canada) who prepared the study products. The main interaction between blinded and unblinded research staff was pickup of study product from pharmacy which had no distinguishing features in containers between the active and placebo. Interactions with study staff mostly took place at participants’ homes. During the time when acute COVID-19 was a greater danger to society, a shift to home visits and remote communication was necessary because research projects were required to be maximally off site from research hospitals to protect staff, hospital clients and the pregnant participants from outbreaks of infection. The trial was registered at ClinicalTrials.gov (NCT03296956).

### Ethics

This study was approved by the CAMH Research Ethics Board (REB#083/2015). Approval of the study by Health Canada was also given, termed as, “no objection” to the project (Clinical Trial Application no. 207773, protocol included, Supplementary Appendix 1). All experiments on human subjects were conducted in accordance with the Declaration of Helsinki and the International Conference on Harmonization's Good Clinical Practice guidelines. All participants provided written informed consent.

### Participants

**Fig. 1:**
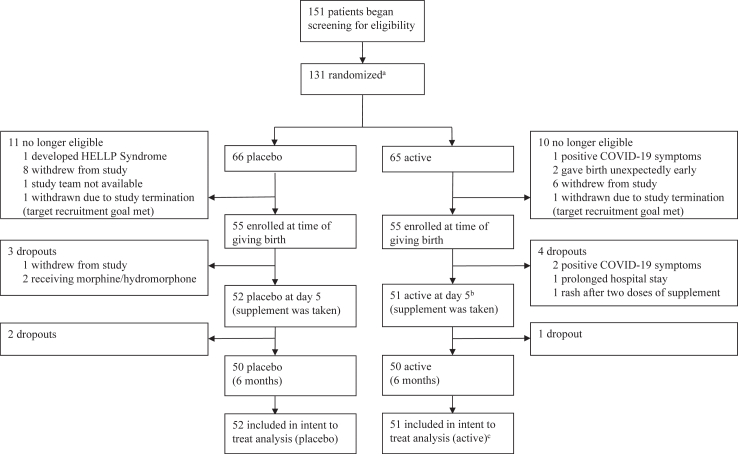
Trial profile: a Randomization took place before giving birth in order to deliver supplement prior to giving birth. b Postpartum blues typically peaks/is present at day 5. c 52 started supplement but one had a rash and did not complete assessments. 52 participants who started taking the supplement had data to include in record of adverse events and 51 participants who completed taking the supplement had data to include in intent to treat analysis.

Between December 1, 2018, and December 25, 2022, 151 participants located within a 3 h car drive of Toronto were recruited by advertisement to begin screening procedures during the third trimester of their pregnancy (Fig. 1). At nighttime of postpartum day 3, 116 participants were eligible and 104 started the supplement with 103 completing the supplement and the primary measures at postpartum day 5. The latter two groups were included in the safety and treatment effect analyses. One-hundred participants completed six month follow-up, ultimately ending July 25, 2023 when the completion of 100 participants through the follow up phase of the study occurred. All participants provided written informed consent.

Main inclusion criteria were age 18–45, self report of good health, pre-pregnancy body mass index of 18.5–40 (kg/m^2^), and normal cardiovascular vital signs. All participants reported being pregnant and therefore of female sex. Main exclusion criteria included history of MDE or other psychiatric illness or substance use disorder in the past 10 years based on the Structured Clinical Interview (SCID) for DSM-5,^20^ smoking cigarettes in the past 5 years, current substance use (screened by urine drug screens at initial screening and at day 5 postpartum, the latter applied as withdrawal criterion), neurological illnesses, autoimmune disease, malignant neoplasm, metabolic disease, or use of medications that could influence mood or are used to treat illnesses associated with changes in mood state. While women with a past history of MDE might benefit from supplementation, our approach was to sample women whose main reason for mood change was due to giving birth rather than history of MDE, which might create more heterogeneity to symptom measures. Since the supplement was not intended as a treatment for PPD, participants were rescreened to exclude MDE prior to taking supplement two weeks before the predicted time to give birth as it was not expected that full MDE symptoms would occur between this timepoint and postpartum day 5.

### Randomization and masking

The ratio of active treatment to placebo was 1:1. Participants were randomized evenly to either active or placebo within randomly chosen blocks of 2, 4, 6, 8 or 10 participants using IBM SPSS version 24 by the CAMH pharmacy. Placebo pills were identical in appearance; whereas placebo pouches and placebo beverage were identical in appearance and taste to their active counterparts (detailed under procedures). To assess success of masking, study participants indicated the degree of their belief that they received active or placebo on a 10 cm visual analogue scale (VAS) at the end of their enrolment.

### Procedures

At the first in person visit completed in their third trimester, participants completed the SCID for DSM-5, the Edinburgh Postnatal Depression Scale (EPDS), the Hamilton Depression Rating Scale, structured questionnaires regarding health and past substance use as well as a urine drug screen to verify current physical and mental health. Two weeks before the estimated due date, participants repeated the mood module of the SCID for DSM-5 and the EPDS to exclude current MDE. At day 3 postpartum, presence of any neonatal or obstetrical complications were reviewed to exclude those with high severity events. After the COVID-19 pandemic began, screening questions to exclude participants with symptoms of COVID-19 were conducted in the evening of day 3 and day 4 postpartum.

Key ingredients of the active dietary supplement included: 2 g of l-tryptophan (Apotex, two 1gram tablets) 10 g of l-tyrosine (Natural Factors, twenty 500 mg tablets), blueberry juice and blueberry extract (Vitablue). On the night of day 3, the morning and evening of day 4, and morning of day 5 postpartum, participants ingested one pouch of active extract or placebo mixed with blueberry juice or placebo. The blueberry juice, blueberry extract and corresponding placebo were prepared by NSF. The placebo pouches matching the blueberry extract contain Shade grape blue powder. The placebo drink was blue through natural colouring with blueberry taste through natural flavouring, but had negligible antioxidant properties and similar sugar level to the active drink. Placebo pills were empty hard gelatin capsules from Capsugel and Lactose monohydrate from Galenova, identical in appearance to encapsulated tryptophan and tyrosine, all prepared by the CAMH pharmacy. Active beverage (weight 369.55 g) included blueberry juice concentrate (9.99%, Milne), filtered water (79.5%, National Sanitation Foundation), natural blueberry flavour (0.75%, Bell Flavours), granulated sugar (5.99%, Caldic) and citric acid (0.1%, Caldic). The pouch, which was added to the beverage just prior to time of ingestion, included Vitablue North American Blueberry Extract (0.55%, Futureceuticals) and granulated sugar (3.12%, Caldic). Placebo beverage (weight 362.55 g) included natural blueberry flavour (1%, Bell Flavours), filtered water (89.11%, National Sanitation Foundation), granulated sugar (5.71%, Caldic), citric acid (0.2%, Caldic) and Shade Bordeaux (0.25%, GNT). The pouch, which was added to the beverage just prior to the time of ingestion included shade grape blue powder (0.28%, GNT) and granulated sugar (3.46%, Caldic). Volumes and ingredient proportions of beverages varied by less than 5%. Taste was identical across the two products as assessed by the National Sanitation Foundation and study personnel at CAMH. ORAC (Oxygen Radical Absorbance Capacity) values of study product from the same batch were assessed prior to use, at 6 months and at 10 months whereupon a new batch was given to verify consistency across batches as well as in comparison to the previous open trial.^19^ Participants were allowed to breastfeed. Phone call reminders were given for the first three doses, and the last dose (inclusive of tyrosine or placebo) was supervised by study staff. Participants were also asked about completion of each previous dose at these timepoints to verify adherence.

### List of outcomes

**Fig. 2:**
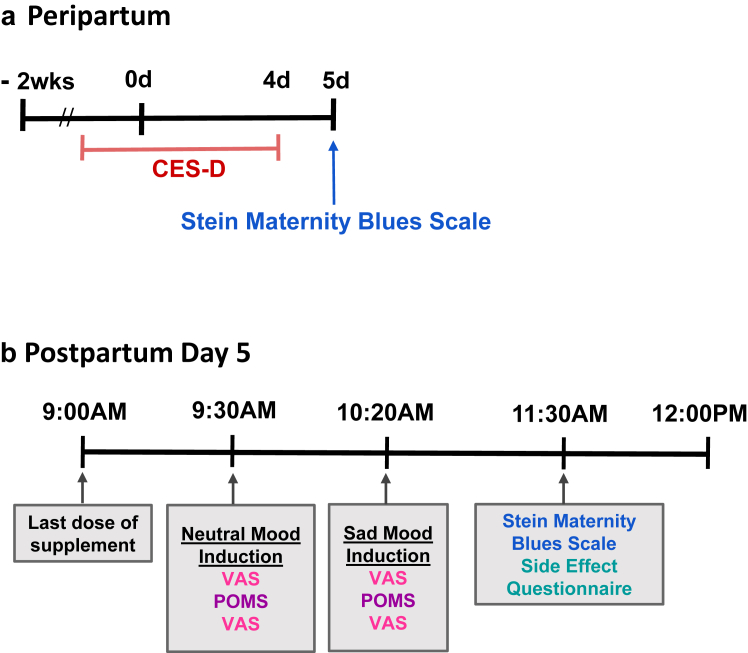
Timeline of key measures in peripartum: CES-D, Center for Epidemiologic Studies Depression Scale (applied to describe days −2 to day 4). VAS, Visual Analogue Scales; POMS, Profile of Mood States.

The primary outcome was change in 10 cm visual analogue scale (VAS) after depressed mood induction as compared to neutral mood induction. The VAS was measured twice approximately 15 min apart, after neutral mood induction and then measured twice again approximately 15 min apart after depressed mood induction, having been studied in the previous open trial of this supplement.^19^ A highly correlated secondary outcome was change in profile of mood states score (POMS) after depressed mood induction as compared to neutral mood induction, with the POMS measured once after each of the same mood inductions as the VAS (Fig. 2). The neutral mood and sad mood induction procedures combine the methods of reading self-referent statements Velten^21^ and music from Clarke^22^ to achieve a consistent response.^19^ As the main aim of the present study was to assess overall severity of postpartum blues after the dietary supplement, an important exploratory outcome was self-report of the Stein Maternity Blues Scale on day 5. The Stein Maternity Blues Scale as a continuous variable was the global measure of postpartum blues because it covers many aspects of postpartum blues, has a clearly defined time for symptom report being the day it is given, is straightforward to administer, is well understood by participants, and has a scale for each item.

Since the second aim was to explore the relationship between the dietary supplement administration and later depressive symptoms, another important exploratory outcome was to assess overall severity of depressive symptoms over the subsequent 6 months with the Center for Epidemiologic Scale for Depression (CES-D). The CES-D was prioritized because it queries a broad range of depressive symptoms, yields similar scores with remote and in person administration, offers a substantial dimensional aspect as a continuous variable, has individual items relevant for healthy states and contains substantial distribution of scale within both healthy and MDE categories. Additional measures recorded included the EPDS and the Beck Depression Inventory (BDI). The EPDS is a measure of risk for presence of a MDE in postpartum, with scores above 10 indicating high likelihood. The BDI is a self-report measure of MDE presence and severity of MDE. Adverse events (AE) were recorded using a structured questionnaire where participants rated severity of symptoms that emerged from mild to severe, on days 3 and 5. AE were considered present if an event post intervention at day 5 was not observed pre-intervention at day 3, or was more severe at day 5 than day 3.

### Statistics

To assess the MIP, a linear mixed effects model examined effect of group (active versus placebo) and induction state (neutral or depressed mood induction) as fixed effects and participant as a random effect, to evaluate the effect of group on change in depressed mood measured by the mean VAS before and after MIP as well as the POMS. The VAS and POMS are continuous variables and repeated measures. To assess overall severity of postpartum blues, a regression analysis with a general linear model was applied, comparing active versus placebo with crying prior to supplement, measured as a question on the CES-D, as a covariate and a categorical variable; with total Stein Maternity Blues Scale score, a continuous variable as the dependent variable. To assess depressive symptoms in the subsequent 6 months, a linear mixed effect model was applied with total CES-D score, a continuous variable, as the dependent variable, evaluating the interaction between time (change in postpartum CES-D from baseline, 10 days, 1 month, 3 months, 6 months postpartum) and group (active versus placebo). No additional covariates were added to this model. Normally distributed variables are described by mean, standard deviation and/or confidence intervals. Variables with a skewed distribution are described by median and interquartile range. For variables with a skewed distribution the median was applied to calculate effect size.

All participants were included in the analyses, but as the original intention was to withdraw cases with MDE prior to supplement administration, analyses of postpartum blues are also presented without two cases who had MDE level symptoms prior to supplement administration. Also, since the CES-D crying score for the week prior to supplement completion had an imbalance with more cases receiving active (n = 4) but not placebo (n = 0), analyses of postpartum blues are also presented with and without this group as a sensitivity analysis. All analyses were carried out using IBM SPSS 25, or SAS version 9.4.

With a sample size of 100 (50 per group), the minimum detectable effect size to achieve 80% power in terms of a difference in change in MIP effect on VAS scores between the diet supplement and the placebo groups is 0.56 (Cohen's d). Effect size was calculated using a two-tailed test and a 0.05 significance level. A data monitoring committee was not used.

### Role of funding source

Approximately 85 per cent of study funding was from CAMH and 15 per cent was from Exeltis. CAMH has a licensing agreement with Exeltis in which CAMH receives funding from Exeltis; and Exeltis will manufacture and distribute the dietary supplement. Exeltis had no role in study design, data collection, data analysis, data interpretation, or writing of the report. Dr. Meyer is the inventor of the supplement. Dr. Meyer was also the principal investigator (PI), qualified investigator for medical supervision of study product administration, wrote first draft of report, designed study, contributed to statistical analyses, led team, and led data management. To address this, there was extensive involvement of two staff statisticians (Dr. Wei Wang and Dr. Sheng Chen) and a study monitor appointed to CAMH independently of Dr. Meyer. The approaches for the main analyses of the study were reviewed and approved (or modified as appropriate) by the statisticians. In addition, study data was provided to a statistician independent of CAMH and Exeltis for statistical analysis (Dr. Mingyang Li), which has been integrated in the manuscript. The study monitor inspected 100% of source documents and 30%–100% of the correctness of their transfer into case report forms as well as transfer to REDCap data storage approximately every 6 months.

## Results

Between January 2019 and December 2022, 151 participants were assessed for eligibility. Of these, 128 were randomized to receive a delivery of active or placebo 1 month prior to giving birth and 116 were eligible at day 3 postpartum (Fig. 1). 104 participants reported starting the supplement on schedule but one participant in the active group had a rash and was withdrawn prior to taking tryptophan or tyrosine. 103 participants began assessment measures and completed intake of the supplement with 51 participants receiving active and 52 participants receiving placebo. All data from participants was included in the study analyses, regardless of their adherence to the supplement or timing of taking the supplement. All 103 cases are included in analyses of symptoms and all 104 cases are included in report of adverse events. During the six month follow up, 3 participants withdrew, 2 after day 10 and 1 after 1 month. Baseline demographic characteristics for active and placebo groups were similar in regards to age, parity, weight, pre-pregnancy body mass index and are described in Table 1.

**Table 1:** Demographic characteristics.

	Supplement (n = 51)	Placebo (n = 52)	All (n = 103)
**Background**			
Age, years	34.20 (3.50)	33.37 (3.80)	33.78 (3.65)
Common Law/Married	49 (96.08%)	49 (94.23%)	98 (95.15%)
Level of education			
≥4-year university	48 (94.12%)	43 (82.69%)	91 (88.35%)
> high school	51 (100.00%)	50 (96.15%)	101 (98.06%)
History of MDE^a^	0	0	0
Ethnicity			
Arab/Middle Eastern	1 (1.96%)	2 (3.85%)	3 (2.91%)
East Asian	2 (3.92%)	9 (17.31%)	11 (10.68%)
South Asian	4 (7.84%)	3 (5.77%)	7 (6.80%)
Black/African-American	1 (1.96%)	3 (5.77%)	4 (3.88%)
Caucasian	38 (74.51%)	28 (53.85%)	66 (64.08%)
Latino/Latina/Hispanic	1 (1.96%)	3 (5.77%)	4 (3.88%)
Other^b^	4 (7.84%)	4 (7.69%)	8 (7.77%)
Employment Status			
Full-time	44 (86.27%)	44 (84.62%)	88 (85.43%)
Part-time	2 (3.92%)	3 (5.77%)	5 (4.85%)
Homemaker	2 (3.92%)	1 (1.92%)	3 (2.91%)
Maternity leave	2 (3.92%)	2 (3.85%)	4 (3.88%)
None	1 (1.96%)	2 (3.85%)	3 (2.91%)
Parity			
1st child	28 (54.90%)	34 (65.38%)	62 (60.19%)
2nd child	16 (31.37%)	17 (32.69%)	33 (32.04%)
More than 2 children	7 (13.73%)	1 (1.92%)	8 (7.77%)
**Pregnancy**			
Enrolled Pre-COVID-19	6 (11.76%)	6 (11.54%)	12 (11.65%)
Multivitamin intake	50 (48.54%)	52 (50.49%)	102 (99.00%)
**Birth**			
Neonatal complications^c^	4 (7.84%)	4 (7.69%)	8 (7.77%)
Male/Female infant sex	28 (54.90%)/23 (45.10%)	22 (42.31%)/30 (57.69%)	50 (48.54%)/53 (51.46%)
**Screening measures**			
BDI screening	3.47 (3.07)	3.88 (2.81)	3.68 (2.94)
Body mass index^d^	23.95 (3.26)	23.51 (3.87)	23.73 (3.58)
**Measures day −2 to 4 before supplement**^e^			
BDI	5.57 (5.45)	5.63 (4.16)	5.60 (4.84)
CES-D crying	0.76 (0.95)	0.52 (0.61)	0.64 (0.80)
CES-D	7.06 (6.88)	7.02 (5.43)	7.04 (6.19)
#MDE (BDI and CES-D ≥ 20)	2 (3.92%)	0 (0.00%)	2 (1.94%)
**Personality characteristics**^f^			
Neuroticism	63.24 (22.58)	66.29 (18.46)	64.78 (20.56)
Extraversion	118.51 (13.64)	117.60 (17.96)	118.05 (15.89)
Agreeableness	136.78 (16.37)	131.13 (15.90)	133.93 (16.30)
Conscientiousness	129.76 (18.02)	130.06 (15.99)	129.91 (16.94)
Openness	120.90 (17.42)	119.31 (15.55)	120.10 (16.44)

MDE, major depressive episode; BDI, Beck Depression Inventory; CES-D, Center for Epidemiologic Studies Depression Scale; CPAP, continuous positive airway pressure; NICU, neonatal intensive care unit.

a History of MDE in past 10 years.

b Including mixed ancestry.

c Three infants had jaundice. Two required CPAP. Two had lung fluid and were admitted to the NICU. One had shoulder dystocia and cephalohematoma.

d Body mass index refers to weight divided by height in square metres (kg/m^2^).

e Relative to giving birth. Data are number (%) or mean (SD).

f From NEO Personality Inventory Revised.

There were group dissimilarities prior to supplement intake regarding symptoms of MDE and crying. Over postpartum day −2 to 4, two participants in the active condition had MDE-level severity symptoms (based on scores ≥20 on the CES-D and BDI) versus zero in the placebo condition, and four individuals in the active condition had CES-D crying scores of 3 versus zero in the placebo group. One single case in the active group had both MDE-level symptom severity and a CES-D crying score of 3 (see Discussion regarding recent literature suggesting these unanticipated differences likely attributable to COVID-19 related stress^23^^,^^24^ and supplementary Figure S1). The dietary supplement is not meant to treat PPD and the study protocol was intended to exclude MDE cases prior to supplement administration. However, the dietary supplement was inadvertently given to the two cases with full MDE symptoms because the study design assumed, based on pre-COVID-19 expectations, that screening two weeks prior to giving birth would be sufficient to exclude MDE cases prior to receiving supplement.

**Fig. 3:**
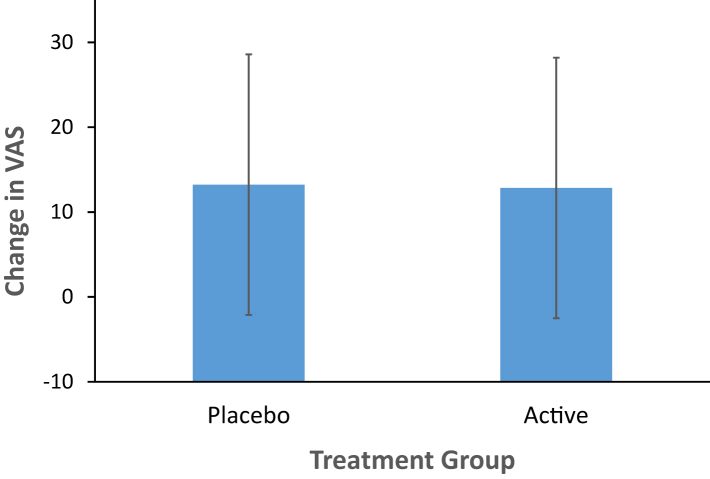
No effect of mood induction procedure on visual analogue scale for depressed mood: VAS, Visual Analogue Scale for Depressed Mood (in mm). Change in VAS shown is mean of third and fourth VAS minus mean of first and second VAS. Error bars represent standard deviation. No significant effect of treatment on change in VAS (mixed effects model, p = 0.90), CI (−6.42, 5.65).

The outcome of the MIP effect on VAS was not significantly different between groups (difference in change score between groups −0.39 mm 95% CI: −6.42 to 5.65 mm, mixed effects model, p = 0.90, Fig. 3). Similarly, independent analysis with general linear regression found no effect of group (p = 0.90). The change in POMS score after neutral mood induction as compared to after MIP, which correlated highly with change score in VAS depressed mood (Spearmann correlation coefficient, r = 0.57, p < 0.001), also showed no significant difference between groups in elevation after MIP procedure between groups (difference in MIP effect on POMS between groups −3.33, 95% CI: −16.61 to 9.96, p = 0.36). Similarly, independent analysis with general linear regression found no effect of group (p = 0.36).

The Stein Maternity Blues Scale scores were lower in the active group (effect size 0.62; median, interquartile range (IQR): active 2.00 (IQR 1, 4); placebo 4.00 (IQR 1.5, 6); regression with general linear model, supplement effect, β coefficient = −1.50 (95% CI: −2.60, −0.40), p = 0.008; effect of CES-D crying before supplement, p = 0.03–0.00000023, latter depending on crying category compared). While the independent statistical analysis indicated that assumptions were met at an acceptable level, there was some skewedness of residuals, largely caused by one case, which, when removed, led to a more significant effect of treatment (p = 0.001). This case was one of the two cases who had MDE level symptoms prior to supplement. Table 2 presents differences between groups, significance and confidence interval for the estimated coefficient with and without the two cases who unexpectedly had MDE level severity symptoms prior to taking the supplement (for histograms, see Supplementary Figure S2). Also, in contrast to a previous open trial conducted prior to COVID-19, it was observed that many participants reported crying in the week prior to taking the supplement as reflected on CES-D crying scores which were significantly more elevated during waves of COVID-19 (exploratory analysis, Wilcoxon Two-Sample Test Statistic = 1247.5, p = 0.037, Supplementary Figure S1). CES-D crying scores prior to supplement were also a strong predictor of postpartum blues at day 5 so they were included in the model. Additionally, as an alternative approach to determining effect size with the median, independent statistical analysis reported the difference in median Stein Maternity Blues Scale found with quantile regression, t = −2.83, p = 0.006.

**Table 2:** Stein maternity blues scales day 5: active versus placebo, sensitivity analysis of sampling.^a^^,^^b^

Cases	Active (n)	Active median stein (IQR)^c^	Placebo (n)	Placebo median stein (IQR)^c^	Estimated coefficient^d^	95% CI of estimated coefficient^d^	Treatment significance^d^
All	51	2.00 (1–4)	52	4.00 (1.5–6)	−1.50	−2.60 to −0.40	0.008
No MDE before supplement^a^	49	2.00 (1–4)	52	4.00 (1.5–6)	−1.72	−2.72 to −0.73	0.0009
No crying score = 3 before supplement^b^	47	2.00 (1–4)	52	4.00 (1.5–6)	−1.50	−2.58 to −0.41	0.007
No MDE or crying score = 3 before supplement^a^^,^^b^	46	2.00 (1–3.75)	52	4.00 (1.5–6)	−1.76	−2.78 to −0.74	0.0009

a MDE, major depressive episode. Original plan was to exclude MDE symptom level cases prior to supplement administration.

b Imbalance in sample such that 4 cases crying almost daily (CES-D crying score = 3) prior to supplement administration happened to be in the supplement group.

c Stein has a skewed distribution so median reported. IQR, Interquartile range (1st quartile-3rd quartile).

d Result from regression analysis with general linear model, crying before supplement covariate as categorical variable, CI, confidence interval.

**Fig. 4:**
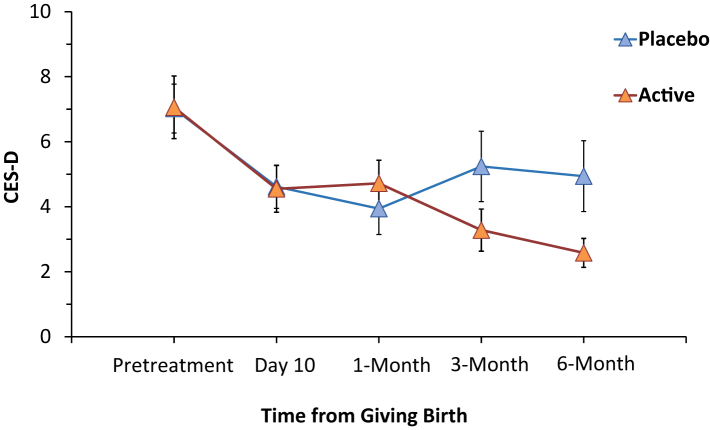
Depressive symptoms over first 6 months postpartum: CES-D, Center for Epidemiologic Studies Depression Scale. Interaction between changes in CES-D over time favoured active supplement (interaction between treatment and time, mixed effects model, p = 0.05; alternatively piecewise regression, p = 0.01). Vertical lines represent standard error.

Regarding change in depressive symptoms from the week prior to supplement to 6 months later, there was a significant interaction between time of CES-D score and active versus placebo reflecting a greater transition to relatively lower CES-D scores in the active group (mixed effects model, F_4__,__397_ = 2.4, p = 0.05, Fig. 4, and Supplementary Figure S3). Independent statistical analysis applying a random intercept and random day effect model with piece-wise day effect, including effects of treatment group, day, day ≥30, day ≥90, day ≥180, and treatment group by day ≥90, found a significant interaction between treatment group and time at 3 months and beyond, t_297_ = −2.59, p = 0.01). A similar trend was observed in change in EPDS scores of an interaction between time of CES-D score and condition of active versus placebo.

Twenty-six and 40 AEs occurred within 25% and 42% of active and placebo cases respectively, and there was a trend that they were less likely to occur in participants in the active group (described in Table 3, Chi-Square, p = 0.06). AEs were likely attributable to being postpartum rather than the protocol, with the possible exception of one rash in the active condition. The reduction of overall AEs in the active condition appeared attributable to less drowsiness and headache in the active condition. There were no serious adverse events.

**Table 3:** Adverse events from days three to five postpartum.

Symptoms	Active no. of adverse events (% of Active Cases)	Placebo no. of adverse events (% of Placebo Cases)
Headache	3 (5.77%)	11 (21.15%)
Drowsy	8 (15.38%)	11 (21.15%)
Nausea	1 (1.92%)	0 (0.00%)
Vomiting	0 (0.00%)	0 (0.00%)
Sweating	3 (5.77%)	2 (3.84%)
Heartburn	2 (3.84%)	0 (0.00%)
Rash	1 (1.92%)	0 (0.00%)
Dry Mouth	4 (7.70%)	4 (7.70%)
Increased heart rate	1 (1.92%)	0 (0.00%)
Dizzy	0 (0.00%)	2 (3.84%)
Confusion	0 (0.00%)	2 (3.84%)
Muscle pain	1 (1.92%)	3 (5.77%)
Restlessness	2 (3.84%)	5 (9.62%)

Data are n (%).

Twenty-six and 40 adverse events occurred in 25% and 42% of supplement and placebo cases respectively (Chi-Square, presence of at least one adverse event in participant, supplement versus placebo group, trend towards less in supplement, p = 0.06). Some participants had more than one adverse event.

Mean scores on the VAS for belief that participants received active versus placebo at the end of the study were similar between treatment groups and non-significant (Wilcoxon Two-Sample Test Statistic = 2670.5, p = 0.31). There was no significant association between belief that the supplement would be effective prior to receiving supplement and reduction in MIP effect on VAS for depression at day 5, reduction in day 5 Stein Maternity Blues Scale, or lower CES-D score at 6 months postpartum.

## Discussion

The main negative finding was no effect of the dietary supplement on severity of depressed mood induction. An important positive finding was that the dietary supplement was associated with less postpartum blues with an effect size of 0.6, which is larger than other interventions in the PPD supplement field.^25^, ^26^, ^27^ Additional findings were less adverse events in those taking the active supplement and that over the following six months, those in the supplement condition shifted towards less depressive symptoms, all of which may be related events. The potential influence of COVID-19 on findings, including the negative finding on MIP, should also be considered.

In the present study, it is plausible that most adverse events are attributable to recently giving birth and that the tendency towards lesser frequency of adverse events with active supplement, might reflect a reduction of postpartum blues symptoms. For example, in the active condition there was less drowsiness and less headache which could correspond to the low energy and headache of postpartum blues. Irrespective of the strategy of compensating for elevated MAO-A level, the individual ingredient of l-tryptophan is known to often assist sleep,^17^ which when better is associated with better energy. l-tyrosine has been shown to enhance cognitive performance during psychological stressors including those that cause physical discomfort and pain,^15^^,^^16^^,^^18^ so collectively the effects of tryptophan and tyrosine could reduce perceived problems of low energy and headache.

Cost-effectiveness of this intervention can be considered in comparison to other prevention approaches for PPD such as peer support, counselling, educational programs, social support, cognitive-behavioural therapy, motivational interviewing, supportive care, mindfulness with or without mobile app support, and antidepressants.^28^^,^^29^ Due to cost and scarcity, and in the case of antidepressants potential adverse events and/or undesirability, with the possible exception of some apps, most of these interventions are only available to women at high risk for MDE. In contrast the supplement in the present study could be available to women at low or high risk for MDE. Clear instructions regarding use and a straightforwardly ingestible version of the supplement would aid generalizability. However, it should be noted that therapy interventions could be blended with the supplement regimen, a possible direction for future clinical trials.

Several limitations were present. First, now known in hindsight, COVID-19 is associated with greater depressive symptoms in postpartum,^23^^,^^24^ plausibly due to additional stresses, such as fear of the baby or mother getting COVID-19 which has been identified as a predictor of depressive symptoms^24^; and an additional concern for participants in the present study was fear of not having a partner present during childbirth due to hospital rules excluding a partner with a symptom of COVID-19. This may account for the two cases not having MDE level symptoms two weeks prior to giving birth yet then having MDE symptoms over −2 to day 4 postpartum; and similarly the high prevalence of crying spells occurring during waves of COVID-19. Also, given the unexpected high prevalence of crying spells reported at the time of birth prior to supplement intake and its strong relation to later severity of postpartum blues, the crying spells rating of the CES-D was applied as a covariate. However this was not planned prior to the trial as COVID-19 emerged during the trial. Another limitation was that the MIP had limited effect compared to a previous study.^19^ Several reasons for this are speculated, such as lack of adaptation of the procedure from the hospital setting to home, where level of comfort associated with at-home administration may have reduced induction of sad mood, environmental distractions may have interfered with steady mood state, or participants may have experienced fear of contracting COVID-19 from the visiting assessment team who were wearing N95 masks and sitting on plastic sheets. While placebo effect and expectancy bias are considerations, there was no evidence that greater participant belief of receiving supplement or greater belief that supplement would be effective was associated with a lesser reduction in VAS change after the MIP.

It may seem conceptually unlikely that a dietary intervention completed at day 5 is associated with less depressive symptoms 6 months later, but the link between severe postpartum blues in early postpartum and presence of depression symptoms several months later is well established. For example, studies from O'Hara and colleagues, Adewuya, and Hannah et al. report consistent results of high level of postpartum blues being associated with 4 fold, 10 fold and 85 fold greater risk of PPD one and a half to two and a half months later.^1^, ^2^, ^3^ In the present study, severity of postpartum blues at day 5 was a strong predictor of depressive symptoms over the following 6 months (mixed effects model, effect of day 5 Stein Maternity Blues Scale on interaction of CES-D and time, p < 0.001). As to how countering effects of elevated MAO-A protein could have longer lasting benefit, preventing monoamine deficits could be helpful since monoamine depletion, whether by reduction of serotonin precursor tryptophan,^8^ inhibition of tyrosine hydroxylase through administration of α-methyl-p-tyrosine^9^ or removal of all three by disruption of vesicular storage via reserpine^10^ leads to depressive syndromes, with the latter occurring after substantial time delay. Future studies could consider larger scale randomized double blind placebo controlled trials to assess the impact of the dietary supplement to prevent postpartum depressive symptoms at thresholds for MDE over 6 month follow up, or to evaluate impact on prevalence of MDE through cohort studies in settings where the dietary supplement is broadly in use.

Overall, the effect size of the dietary supplement for reducing postpartum blues was higher than previous report of dietary supplements tested for preventing postpartum blues or PPD. The supplement was also associated with less adverse events as compared to placebo which can be attributed to by less fatigue and headaches possibly reflecting a reduction in postpartum blues. COVID-19 is suspected to have led to unexpected onset of MDE level symptoms and frequent crying spells in participants earlier in the postpartum period than past timeframes, and adapting the MIP to home use combined with the stressful conditions of COVID-19 may have interfered with its performance. The exploratory finding of progressively less depressive symptoms over time to six months follow up in the active condition is a promising direction for further study, and might be related to impact on postpartum blues, which is known to strongly predict later depressive symptoms.

## Contributors

JHM was the principal investigator, qualified investigator for medical supervision of study product administration, wrote first draft of report, designed study, contributed to statistical analyses, led team, led data management. ZW, AS, YD, YK, AS, ND, JP, YW, RW, HR, ZN—contributed to participant recruitment, and data collection. YD—contributed to study design. MIH—provided backup medical supervision of study product administration. AS, WW, SC, RW, HR, CBT, VS—completed statistical analyses. JHM, ZW, AS, AS, ND, JP, YW, VS accessed and verified the underlying data and take responsibility for the integrity of the data and the accuracy of the data analysis. All authors had full access to all the data in the study. All authors contributed to critical review and editing of the manuscript and had final responsibility for the decision to submit for publication. All authors read and approved the final manuscript.

## Data sharing statement

The study protocol will be attached as appendix 1 at the time of publication. The deidentified clinical trial data will be stored on the CAMH research server and available upon reasonable request to the corresponding author via email subsequent to the time of publication.

## Declaration of interests

JHM is the inventor on patents for the dietary supplement and there is an agreement between CAMH and Exeltis for the latter to manufacture, and distribute the dietary supplement. JHM also has patents for blood biomarkers in mood disorders to predict neuroinflammation, elevated MAO-B level and elevated MAO-A level in the brain. JHM has received operating grant funding from Exeltis and Sanofi in the past 2 years. MIH receives research grants from the Canadian Institutes of Health Research, CAMH Foundation, University of Toronto, COMPASS Pathfinder; stipend from Society of Biological Psychiatry; payment or honoraria from the American Society of Clinical Psychopharmacology and American College Health Association; consulting fees from Wake Network Inc. and stock options in Mindset Pharma Inc.

## Appendix A Supplementary data

Supplementary Section

Supplementary Figures

Study Protocol
