# Supplementary material for: Dietary supplement for mood symptoms in early postpartum: a double-blind randomized placebo controlled trial
Source: eClinicalMedicine. 2024 Apr 10;71:102593. doi: 10.1016/j.eclinm.2024.102593 (PMC11133796; doi:10.1016/j.eclinm.2024.102593)
Supplement: Supplementary Section [file mmc1.docx]

**Supplementary Appendix 1**

Supplement to: Meyer JH, Wang Z, Santhirakumar A, et al. Dietary supplement for mood symptoms in early postpartum: a double-blind randomized placebo controlled trial

**Supplementary Introduction**

Previous study applied [^11^C]harmine positron emission tomography (PET) to evaluate monoamine oxidase A total distribution volume (MAO-A V_T_), an index of MAO-A level, during postpartum days 4 to 6 as compared to healthy women not recently pregnant^1^. Harmine binds to the functional segment of MAO-A protein^2^. [^11^C]Harmine has excellent imaging qualities with high brain uptake; high affinity (Ki, 2nM) and high selectivity for MAO-A; and lack of brain penetrant radiolabelled metabolites^3,4^. Among investigations in rodents, cell lines and non-human primates, inverse relationships between estrogen changes and transient elevations in monoamine oxidase A (MAO-A) level, mRNA and activity are consistently reported^5-9^. In the first few days postpartum there is a several hundred fold decline in estrogens in humans, which attenuates to a substantial extent at approximately day 4 to 6 postpartum^10,11^. On average, across postpartum days 4 to 6, a 43% greater MAO-A V_T_ was reported throughout grey matter regions sampled in women recently postpartum as compared to women not recently pregnant. Grey matter regions with this change included affect modulating structures like prefrontal and anterior cingulate cortex, emotion processing regions like hippocampus and thalamus and the midbrain which includes monoaminergic nuclei that control sleep-wake rhythms. This finding was most pronounced on day 5 as compared to days 4 and 6, such that the cases on day 5 were over 50% elevated as compared to healthy women not recently pregnant ^1^. The spatial distribution of elevated MAO-A V_T_ in the brain included all grey matter regions with substantial MAO-A protein as indicated with a voxel based analysis^1^. MAO-A level tends to be correlated highly with MAO-A activity in brain ^12,13^ so elevations in MAO-A V_T_ have implications for monoamine metabolism and generation of hydrogen peroxide. Measures indicative of elevated MAO-A level, particularly in prefrontal and anterior cingulate cortex, are associated with several depressive syndromes such as major depressive episodes (MDE) of major depressive disorder^14,15^, postpartum depression^16^, sad mood during early withdrawal from alcohol and cigarette use disorders^17,18^, and crying during perimenopause^19^.

There is substantial evidence for brain penetration of monoamine precursors and antioxidants. Both l-tryptophan and l-tyrosine are transported across the blood brain barrier by a large neutral amino acid transporter. The transport for these individual amino acids is related to their availability relative to other large neutral amino acids; which is substantially increased after oral supplementation. Oral l-tryptophan at a dose of 2g raises peak blood plasma level seven fold in women and oral l-tyrosine at a dose of 10mg also raises peak blood plasma level seven fold in women^20,21^. Empirically, oral doses of tryptophan and tyrosine leading to such changes in plasma level of these amino acids also lead to similar changes in cerebrospinal fluid concentration of these amino acids^22,23^, providing good evidence of brain penetration. Anthocyanins are antioxidant compounds found in the blueberry extract and blueberry juice. There are a number of preclinical reports of anthocyanins being detected in brain after regular oral intake^24,25^. In humans, oral intake is detectable in blood plasma, with arguably the most definitive study demonstrating that radiolabelled [^13^C] cyanidin-3-glucoside had a bioavailability of 13%^26^. Oral administration of [^14^C]anthocyanins to rodents indicate that approximately 2% dose per g of anthocyanins are subsequently found in brain tissue^27^.

**Supplementary Discussion**

Another limitation is that it would have been ideal to demonstrate the MAO-A level of the study population as it may be expected that those with the highest level of MAO-A in brain regions that influence symptoms of postpartum blues would benefit most from the supplement. This additionally would provide a comprehensive assessment of the relationship of postpartum blues with MAO-A level. However such an approach was impractical because, during times when COVID-19 was considered particularly harmful to functioning of hospitals, access to hospital research resources was limited and the currently validated method to measure MAO-A level in brain is with arterial sampling and positron emission tomography^1^, already a scarce resource. To date, surrogate measures indicative of MAO-A level in the brain have not been established.

**References**

1. Sacher J, Wilson A, Houle S, et al. Elevated Brain Monoamine Oxidase A Binding in Early Postpartum. *Arch Gen Psychiatry* 2010; **May**(67(5)): 468-74.

2. Son SY, Ma J, Kondou Y, Yoshimura M, Yamashita E, Tsukihara T. Structure of human monoamine oxidase A at 2.2-A resolution: the control of opening the entry for substrates/inhibitors. *Proc Natl Acad Sci U S A* 2008; **105**(15): 5739-44.

3. Ginovart N, Meyer JH, Boovariwala A, et al. Positron emission tomography quantification of [11C]-harmine binding to monoamine oxidase-A in the human brain. *J Cereb Blood Flow Metab* 2006; **26**(3): 330-44.

4. Bergstrom M, Westerberg G, Langstrom B. 11C-harmine as a tracer for monoamine oxidase A (MAO-A): in vitro and in vivo studies. *Nucl Med Biol* 1997; **24**(4): 287-93.

5. Luine VN, McEwen BS. Effect of oestradiol on turnover of type A monoamine oxidase in brain. *J Neurochem* 1977; **28**(6): 1221-7.

6. Holschneider DP, Kumazawa T, Chen K, Shih JC. Tissue-specific effects of estrogen on monoamine oxidase A and B in the rat. *Life Sci* 1998; **63**(3): 155-60.

7. Gundlah C, Lu NZ, Bethea CL. Ovarian steroid regulation of monoamine oxidase-A and -B mRNAs in the macaque dorsal raphe and hypothalamic nuclei. *Psychopharmacology (Berl)* 2002; **160**(3): 271-82.

8. Smith LJ, Henderson JA, Abell CW, Bethea CL. Effects of ovarian steroids and raloxifene on proteins that synthesize, transport, and degrade serotonin in the raphe region of macaques. *Neuropsychopharmacology* 2004; **29**(11): 2035-45.

9. Ma ZQ, Violani E, Villa F, Picotti GB, Maggi A. Estrogenic control of monoamine oxidase A activity in human neuroblastoma cells expressing physiological concentrations of estrogen receptor. *Eur J Pharmacol* 1995; **284**(1-2): 171-6.

10. Nott PN, Franklin M, Armitage C, Gelder MG. Hormonal changes and mood in the puerperium. *Br J Psychiatry* 1976; **128**: 379-83.

11. O'Hara MW, Schlechte JA, Lewis DA, Wright EJ. Prospective study of postpartum blues. Biologic and psychosocial factors. *Arch Gen Psychiatry* 1991; **48**(9): 801-6.

12. Saura J, Kettler R, Da Prada M, Richards JG. Quantitative enzyme radioautography with 3H-Ro 41-1049 and 3H-Ro 19- 6327 in vitro: localization and abundance of MAO-A and MAO-B in rat CNS, peripheral organs, and human brain. *J Neurosci* 1992; **12**(5): 1977-99.

13. Tong J, Meyer JH, Furukawa Y, et al. Distribution of monoamine oxidase proteins in human brain: implications for brain imaging studies. *J Cereb Blood Flow Metab* 2013; **33**(6): 863-71.

14. Meyer JH, Ginovart N, Boovariwala A, et al. Elevated monoamine oxidase a levels in the brain: an explanation for the monoamine imbalance of major depression. *Arch Gen Psychiatry* 2006; **63**(11): 1209-16.

15. Johnson S, Stockmeier CA, Meyer JH, et al. The Reduction of R1, a Novel Repressor Protein for Monoamine Oxidase A, in Major Depressive Disorder. *Neuropsychopharmacology* 2011.

16. Sacher J, Rekkas PV, Wilson AA, et al. Relationship of monoamine oxidase-A distribution volume to postpartum depression and postpartum crying. *Neuropsychopharmacology* 2015; **40**(2): 429-35.

17. Bacher I, Houle S, Xu X, et al. Monoamine oxidase a binding in the prefrontal and anterior cingulate cortices during acute withdrawal from heavy cigarette smoking. *Arch Gen Psychiatry* 2011; **68**(8): 817-26.

18. Matthews BA, Kish SJ, Xu X, et al. Greater monoamine oxidase a binding in alcohol dependence. *Biol Psychiatry* 2014; **75**(10): 756-64.

19. Rekkas PV, Wilson AA, Lee VW, et al. Greater monoamine oxidase a binding in perimenopausal age as measured with carbon 11-labeled harmine positron emission tomography. *JAMA Psychiatry* 2014; **71**(8): 873-9.

20. Dowlati Y, Ravindran AV, Maheux M, Steiner M, Stewart DE, Meyer JH. No effect of oral tyrosine on total tyrosine levels in breast milk: implications for dietary supplementation in early postpartum. *Arch Womens Ment Health* 2014; **17**(6): 541-8.

21. Dowlati Y, Ravindran AV, Maheux M, Steiner M, Stewart DE, Meyer JH. No effect of oral L-tryptophan or alpha-lactalbumin on total tryptophan levels in breast milk. *Eur Neuropsychopharmacol* 2015; **25**(6): 779-87.

22. Eccleston D, Ashcroft GW, Crawford TB. Effect of tryptophan administration on 5HIAA in cerebrospinal fluid in man. *J Neurol Neurosurg Psychiatry* 1970; **33**(2): 269-72.

23. Growdon JH, Melamed E, Logue M, Hefti F, Wurtman RJ. Effects of oral L-tyrosine administration on CSF tyrosine and homovanillic acid levels in patients with Parkinson's disease. *Life Sci* 1982; **30**(10): 827-32.

24. Kalt W, Blumberg J, McDonald J, et al. Identification of Anthocyanins in the Liver, Eye and Brain of Blueberry-Fed Pigs. *J Agric Food Chem* 2008; **56**: 705-12.

25. Andres-Lacueva C, Shukitt-Hale B, Galli RL, Jauregui O, Lamuela-Raventos RM, Joseph JA. Anthocyanins in aged blueberry-fed rats are found centrally and may enhance memory. *Nutr Neurosci* 2005; **8**(2): 111-20.

26. de Ferrars RM, Czank C, Zhang Q, et al. The pharmacokinetics of anthocyanins and their metabolites in humans. *Br J Pharmacol* 2014; **171**(13): 3268-82.

27. Janle EM, Lila MA, Grannan M, et al. Pharmacokinetics and tissue distribution of 14C-labeled grape polyphenols in the periphery and the central nervous system following oral administration. *J Med Food* 2010; **13**(4): 926-33.
