# Supplementary material for: Dietary supplement for mood symptoms in early postpartum: a double-blind randomized placebo controlled trial
Source: eClinicalMedicine. 2024 Apr 10;71:102593. doi: 10.1016/j.eclinm.2024.102593 (PMC11133796; doi:10.1016/j.eclinm.2024.102593)
Supplement: Supplementary Figures [file mmc2.pdf]

Supplementary Figure 1. Effect of COVID Waves[a] and CES-D Crying[b]

Fig 1a. Daily New Cases of COVID in Region

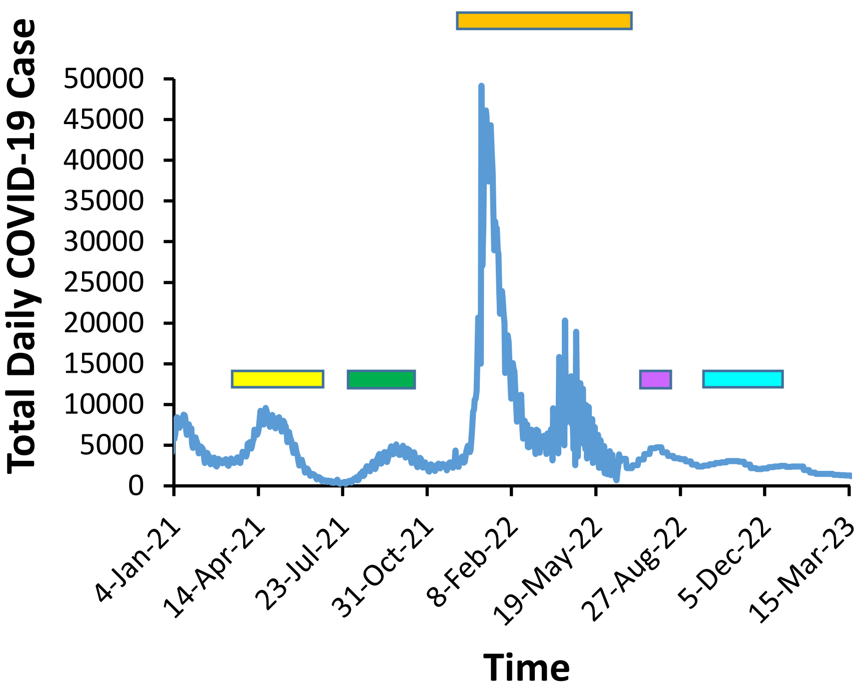

Fig 1b. CES-D Crying Scores[b] and Day 5 Postpartum Dates

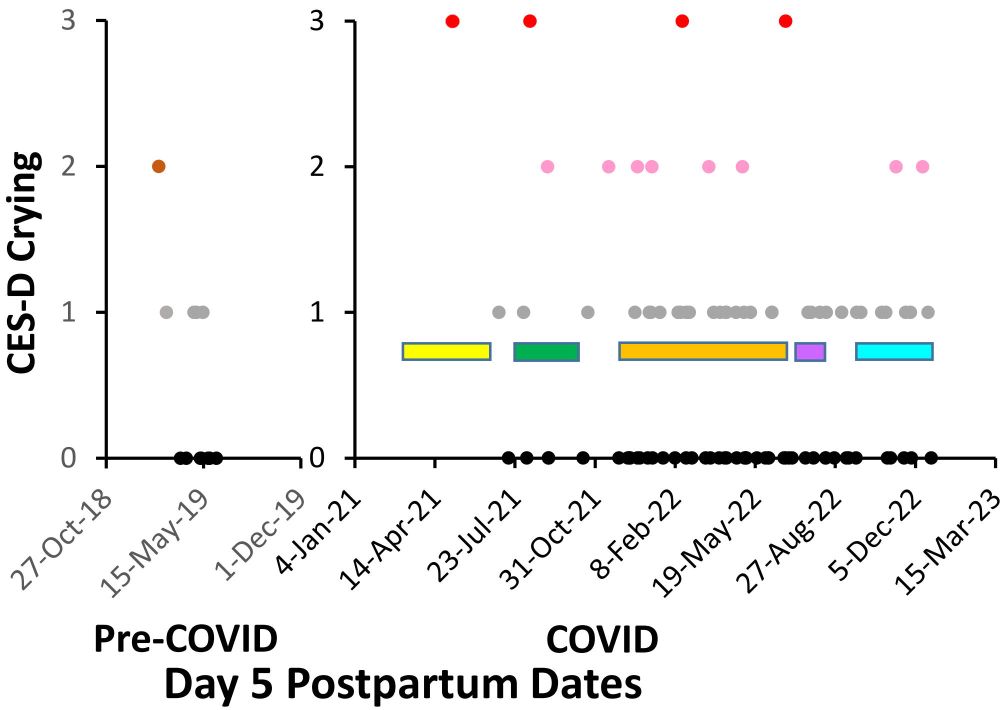

[a] Defined as weekly increase in cases of ~30%, and subsequent elevations until there is a decline in cases to levels reaching within 10% to 20% of the initial increase in cases.

- Wave 1 - Mar 15, 2021 - Jul 3, 2021
- Wave 2 - Aug 1, 2021 - Oct 24, 2021
- Wave 3 - Dec 15, 2021 - June 26, 2022
- Wave 4 - Jul 8, 2022 - Aug 14, 2022
- Wave 5 - Sep 24, 2022 - Dec 25, 2022

[b] CES-D=Center of Epidemiologic Studies Depression Scale. CES-D Crying Score=crying frequency on CES-D item.
Waves of COVID-19 (during study period of March 15, 2021 to December 25, 2022) identified on Supplementary Figure 1a by colour bars also shown on Supplementary Figure 1b. There was a greater level of CES-D crying during COVID waves (Wilcoxon Two-Sample Test Statistic=1247·5, p=0·037).

Supplementary Figure 2. Stein Maternity Blues Scale Scores by Treatment Group

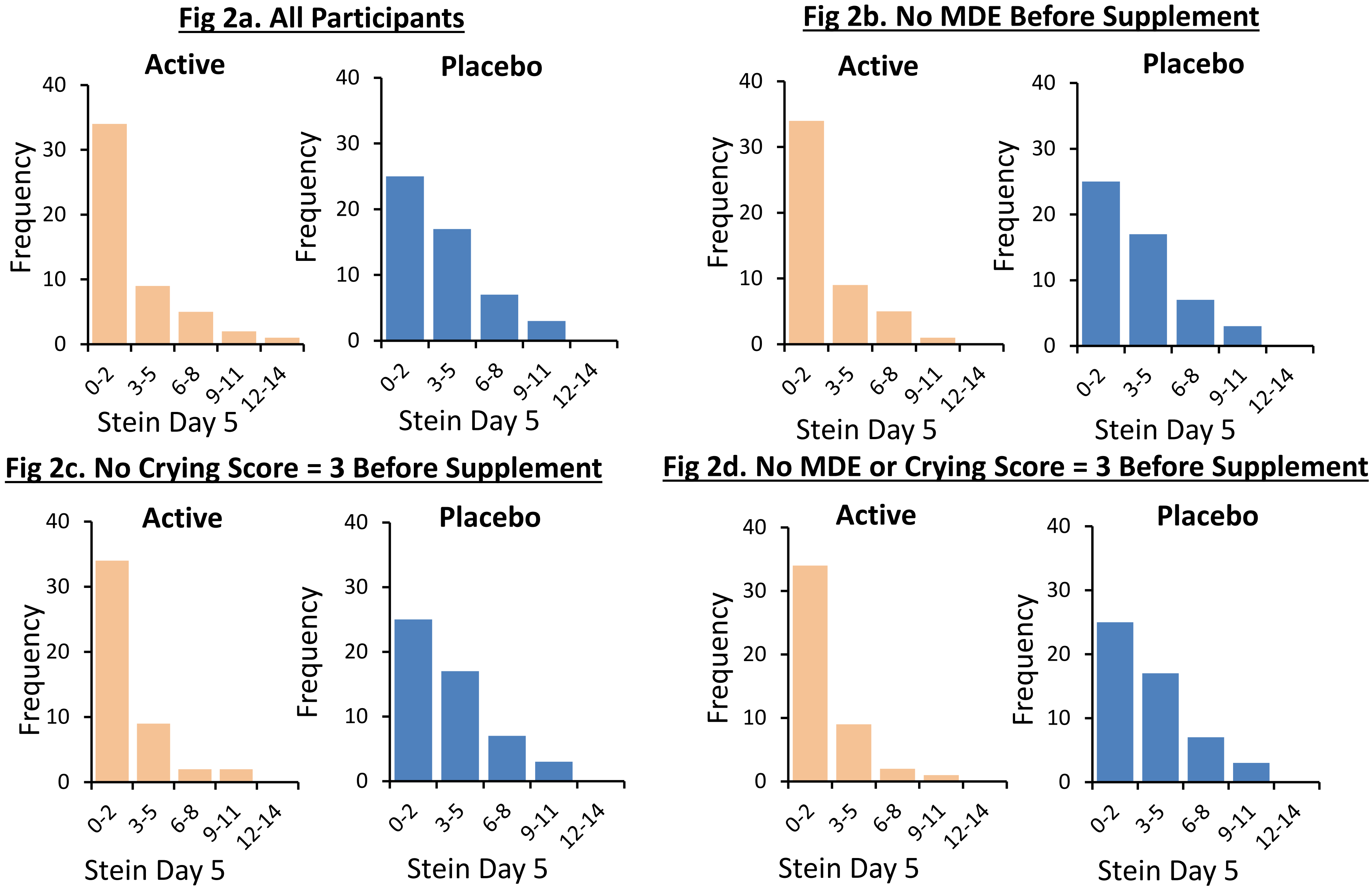

MDE=Major Depressive Episode. Stein refers to scores on the Stein Maternity Blues Scale.
Stein Maternity Blues Scale scores lower in treatment groups (regression analysis with general linear model, crying before supplement covariate, effect of treatment: 2a, *p*=0·008; 2b, *p*=0·0009; 2c, *p*=0·007; 2d, *p*=0·0009).

Supplementary Figure 3. CES-D Scores Over Time in Active and Placebo Groups[a]

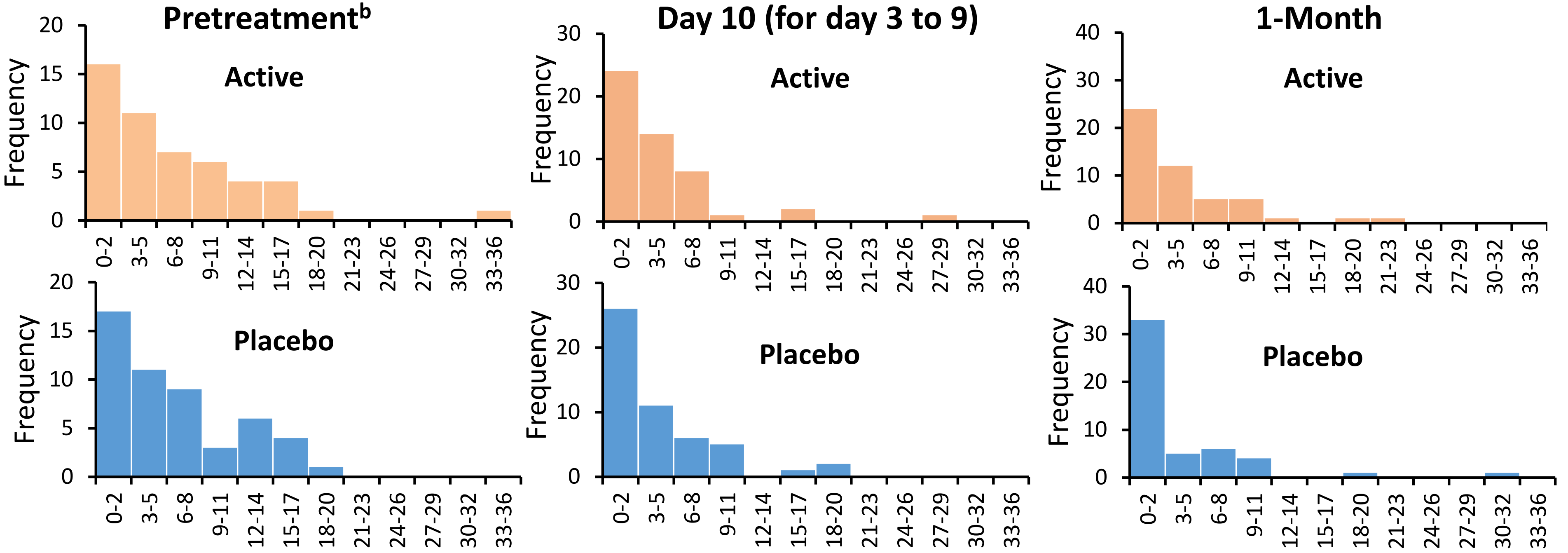

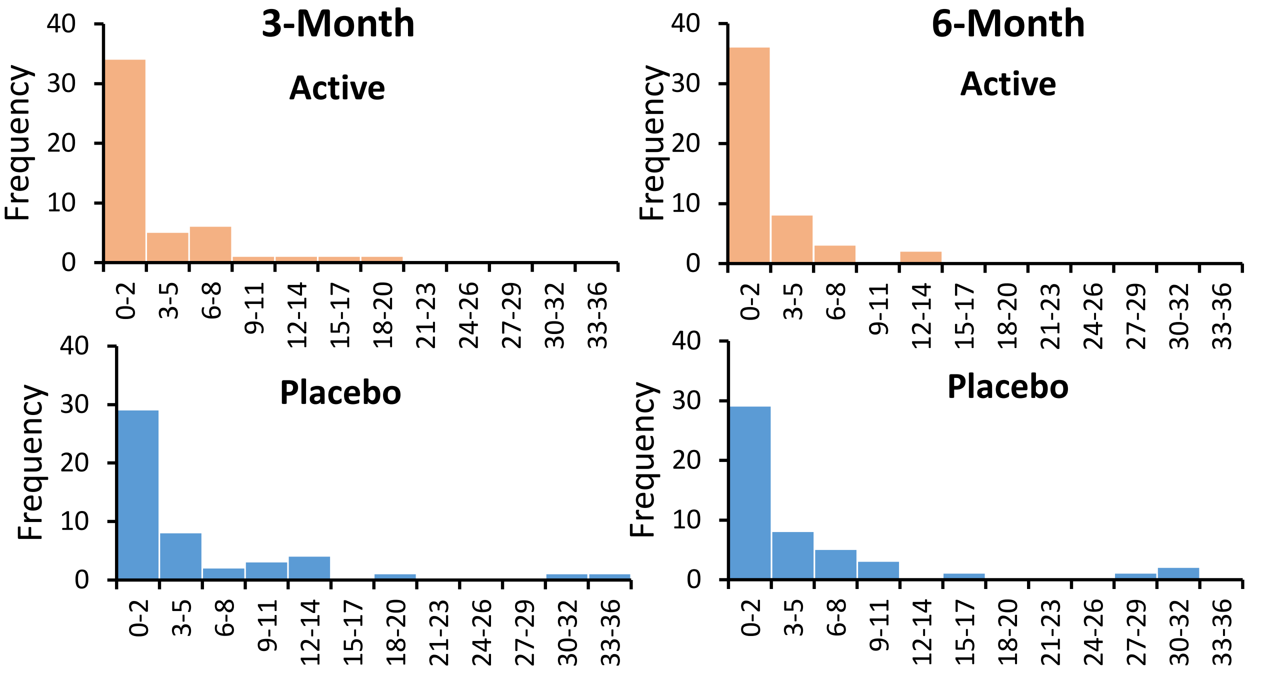

CES-D=Center for Epidemiologic Studies Depression Scale.

[a] X-axis of each graph indicates range of CES-D Score.

[b] CES-D describes the week of day -2 to day 4 postpartum.

Includes all cases who took supplement (n=103; 100 completers).

Interaction between changes in CES-D over time favoured active supplement (mixed effects model, interaction between treatment and time, p=0·05).
