# Supplementary material for: Dietary supplement for mood symptoms in early postpartum: a double-blind randomized placebo controlled trial
Source: eClinicalMedicine. 2024 Apr 10;71:102593. doi: 10.1016/j.eclinm.2024.102593 (PMC11133796; doi:10.1016/j.eclinm.2024.102593)
Supplement: Study Protocol [file mmc3.pdf]

A Dietary Supplement for Mood Symptoms in Early Postpartum: A Double Blind Randomized Placebo Controlled Trial

1. Overview

Postpartum depression (PPD) is the most common complication of childbearing with a prevalence rate of approximately 13%[1]. Currently the World Health Organization ranks major depressive disorder as the second leading cause of death and disability in women (with Canada being affected similarly compared to other places)[2]. Postpartum depression has impact upon child care, return to work, and elevates the risk of future major depressive episodes (MDE) [3, 4]. Risk for PPD is influenced by clinical history, as well as psychological, social and biological factors [5-11]. Severity of postpartum blues is predictive of risk for postpartum depression, so reducing severity of postpartum blues can be a strategy for preventing PPD, based upon a biological model of excessive monoamine metabolism.

Our biological model for postpartum blues is as follows: With the loss of placenta, there is a 100 to 1000 fold drop in estrogen levels in the first three days postpartum[6, 7, 12]. Declines in estrogen are associated with elevations in monoamine oxidase A (MAO-A) in cell lines, and animal models[13-19]. We have previously completed the first study of brain MAO-A levels in the early postpartum period: Our data in a positron emission tomography study in humans shows a 43% elevation in MAO-A binding throughout all brain regions during days 4 to 6 postpartum. MAO-A metabolizes serotonin, norepinephrine and dopamine and MAO-A creates oxidation[20]. Depletion of these neurochemicals is associated with sad mood[21-25]. Greater levels of MAO-A in the prefrontal and anterior cingulate cortex occur during MDEs and postpartum MDEs, as well as during high risk periods for the condition, including prior to recurrence of illness [21-23, 26-28].

To reduce the intensity of postpartum blues, a dietary supplement was created to counter the effects of elevated monoamine oxidase A (MAO-A). This dietary supplement was composed of tryptophan, tyrosine, blueberry juice and blueberry extract. Tryptophan is the amino acid precursor to serotonin and tyrosine is the amino acid precursor to norepinephrine and dopamine, important neurochemicals metabolized by MAO-A in the brain. Tryptophan and tyrosine were intended to replace MAO-A metabolism of serotonin, norepinephrine and dopamine. The anthocyanins in blueberries have some properties that suggest they should be brain penetrant, such as small molecular weight, and it has been reported that anthocyanins cross the blood brain barrier in rodents [29-33].

In previous development, we demonstrated that the tryptophan and tyrosine ingredients, which were greater than dietary levels, do not affect their total concentration in breast milk[34, 35]. This happens because most of the amino acids in breast milk (about 98%) are in fixed protein chains that are unaffected by oral amino acid intake. In another previous development, we also brought the depressed mood induction procedure (MIP) into the assessment of postpartum blues[36]. For this MIP, sad statements are read and sad music is heard, and assessments of mood state are administered before and after. Most importantly, in our recent open-label trial which is almost completed we found that this dietary supplement containing 2 grams tryptophan, 10 grams tyrosine, blueberry juice and blueberry extract was able to reduce the intensity of postpartum blues as measured by mood scores on Visual Analog Scale (VAS) after sad MIP compared to those not receiving any supplements. Although the effect size of this study was large (5.4), there remains the question of the contribution of the placebo effect unless it is studied in a double blind randomized placebo controlled trial (RCT).

Creating a dietary supplement to prevent PPD is presumably a multistep process. One key step of the process is to develop a component that prevents postpartum blues. If the proposed study has a positive result, it would definitively support use of the supplement ingredients to prevent postpartum blues and would become a key component of the eventual dietary supplement to prevent PPD.

Hypothesis:

Sadness after depressed mood induction during postpartum blues will be attenuated in those receiving the dietary supplement (tryptophan, tyrosine and blueberry juice/extract) compared to placebo.

Methods:

One hundred forty pregnant women who are healthy and have no history of MDE will be recruited, typically during their third trimester and it is expected 100 subjects to complete the study. They will be randomized in a double blind manner within blocks of 2, 4, 6, 8 or 10 subjects. Women will start taking the dietary supplement at 4 time points (as indicated in table 2) from day 3 postpartum to day 5 postpartum.

Using a supplement with the same ingredients as a recent open trial, the supplement consists of 2 grams tryptophan, 10 grams tyrosine and blueberry juice/extract. The only difference is that we propose to give a similarly equivalent but non-identical blueberry juice and extract. The volume of the blueberry extract is 2 grams. The appearance and taste of the placebo is indistinguishable from active treatment (as verified by independent manufacturer and investigator visual and taste testing during placebo product development).

Sad mood induction will be done based on the Velten[37] Mood Induction Procedure (MIP). followed by presented with a piece of sad music, from work by Clark et al [38]. Clinical rating measures of mood and symptoms of depression will be assessed before and after MIP.

Importance:

There is no widespread commonly applied method to prevent PPD, and, to our knowledge, there is no dietary supplement that is promising for reducing postpartum blues with the exception of the one we propose to investigate in this study. A dietary supplement to reduce likelihood of PPD would have exceptional high impact in benefiting society, given that PPD is the most common complication of childbearing at a prevalence rate of 13%[1].

2. Background

2.1. Importance of Postpartum Depression

Overview of Mood Disturbances in the Postpartum Period: There are three types of mood disturbances involving sadness in the postnatal period. One is the "baby blues" or maternity blues which is common (occurring up to 75% of the time) and transient finishing within the first week postpartum [5, 6]. The second is a major depressive episode (MDE) with postpartum onset ("postpartum depression") which is defined as a MDE that occurs within the first four weeks after delivery [39]. **Greater severity of the common healthy range baby blues/maternity blues is associated with greater risk for subsequent clinical level postpartum depression[7, 9, 40, 41].** The third is postpartum psychosis (which may be accompanied by depressive symptoms). The third condition is rare occurring in 0.1 to 0.2 per cent of deliveries and is strongly associated with presence of bipolar disorder [42, 43].

Importance of Clinical Level Postpartum Depression (MDE with Postpartum Onset): Postpartum depression (PPD), defined as a MDE starting within the first year after giving birth, is highly impactful, being the most common complication of childbearing with a prevalence rate of 13 %[11, 44-48]. Consistent with this, the World Health Organization has identified major depressive disorder (MDD) as the leading cause of death and disability worldwide[49]. A MDE in early postpartum can lead to serious consequences: During the first postpartum year, presence of psychiatric illness is associated more than a 10 fold risk of suicide [50] and women who have experienced MDE with postpartum onset are at greater risk of having further MDE both following future deliveries and at times unrelated to childbirth [10, 51-54]. Also, MDE with postpartum onset may last for years[55, 56], and has deleterious effect on infant motor and social development and the health of other children in the family and the partner[5, 45].

Despite the tremendous negative impact of postpartum depression, there are no widespread standard methods for preventing its onset. For high risk cases, options to prevent PPD are interpersonal therapy, cognitive behavioural therapy and use of antidepressants such as selective serotonin reuptake inhibitors (SSRI) [57, 58]. The reality is that in the absence of symptoms prior to delivery, pregnant women are preparing for a new child and are usually not interested in obtaining 12 to 18 one hour sessions of therapy (needed for interpersonal or cognitive behavioural therapy) or taking medication for a condition that has not yet manifested [59]. The need for a new method to prevent PPD has been recognized as reflected by recent study that investigated interpersonal peer support via phone calls to prevent PPD but the actual rate of postpartum depression at the time-point assessed in enrollees was the virtually the same in the group receiving the peer support as the control condition[60]. Hence there is still a real need to develop prevention strategies prior to onset of symptoms that are highly feasible[58].

The etiology of PPD is likely multifactorial as social and biological factors have been identified, but if one can prevent an important factor in early postpartum, this should reduce the risk of PPD. **In this proposal we will explain why elevated monoamine oxidase A (MAO-A) levels early in postpartum is strongly implicated as an important factor in the onset of PPD and then we make a case for developing a nutritional supplement to counter the effects of elevated MAO-A levels to prevent PPD.** The proposal also includes the steps and studies that have done so far for developing such a dietary supplement.

2.2. Risk Factors for Postpartum Depression

Risk for PPD is influenced by clinical history, as well as social and psychological factors. The three elements of clinical history which are probably the strongest factors are a previous history of major depressive episodes (in postpartum or at other times), depressive symptoms during pregnancy and severity of postpartum blues [7, 40, 41, 61]. In addition to psychological factors of a clinical history of MDE, neuroticism has been associated with greater likelihood for symptoms of postpartum depression [9]. Significant social factors include lack of a confidant, marital conflict, and possibly social class, lack of social support, and level of current stressors [5, 6, 8, 9, 44]. Some, but not all reports have implicated obstetrical complications as a risk factor for postpartum depression [9, 41, 62]. Women with postpartum blues have up to 4x the risk of developing PPD compared to women with no symptoms of postpartum blues.

2.3. Monoamine Oxidase-A and Its Relationship to Monoamine Metabolism

Monoamine Oxidase A (MAO-A) is an important enzyme found on the outer mitochondrial membrane in neurons and glia in the brain[63-65]. MAO-A levels are proportional to MAO-A activity in brain[66-68]. **Key functions in relation to mood dysregulation are related to its functions to metabolize serotonin, norepinephrine, dopamine which are outlined as follows:** Serotonin is a high affinity substrate for MAO-A [69-72] and MAO-A is detectable in serotonin releasing neurons [73, 74]. MAO-A clearly influences extracellular serotonin because administration of MAO-A inhibitors increases extracellular serotonin from 20 to 200 per cent, depending upon drug, dose and region [75-80]. This has been found in at least six separate studies and across four different MAO-A inhibitors (clorgyline, moclobemide, harman, befloxatone) [75-80] and the finding was present in a variety of brain regions including prefrontal cortex, hippocampus, and superior raphe nuclei. In these paradigms it is often demonstrated that brain 5-HIAA is reduced [76, 77, 79]. Moreover, extracellular serotonin is also raised substantively (100-200%) in prefrontal cortex, hippocampus and superior raphe nuclei in the knockout model of MAO-A [81]. Norepinephrine is a high affinity substrate for MAO-A [72, 82] and MAO-A is easily detectable in cells that synthesize norepinephrine [63, 73, 74, 83]. Under conditions of MAO-A inhibition, extracellular norepinephrine is increased in prefrontal cortex as well as hippocampus (regions assayed in the studies) [84-86] which argues that MAO-A has a substantial role in controlling extracellular norepinephrine. Dopamine is also high affinity substrate for MAO-A [69-71] and administration of MAO-A inhibitors increases extracellular dopamine in striatum under baseline conditions as well as during precursor loading paradigms [87-94]. We are aware of a couple of reports detecting MAO-A in dopamine synthesizing neurons [74, 95], although it has been postulated that MAO-A outside of dopamine synthesizing neurons is more likely to account for the elevations in extracellular striatal dopamine after MAO-A inhibition [63].

Location of MAO-A: In human brain MAO-A density is homogenously distributed within in most brain structures including cortex, with the highest density in the locus coeruleus, and high density in cortex, striatum, thalamus, with lower density in cerebellar cortex and the lowest density in white matter[63, 68, 96].

2.4. Monoamine Oxidase A and Its Relationship to Major Depressive Disorder

In 2006 we discovered that MAO-A levels were elevated during MDE (published in the Archives of General Psychiatry, the top psychiatric journal at that time). MAO-A DV_S, an index of MAO-A density, was measured in 17 MDE (secondary to major depressive disorder) and 17 healthy subjects with [^{11}C] harmine positron emission tomography. The subjects were otherwise healthy. Depressed subjects had early onset depression (before age 40), were drug free for at least 5 months although most were antidepressant naive. Depressed subjects were aged 18-50, met DSM-IV diagnosis of current major depressive episode (MDE) and major depressive disorder (MDD) verified by SCID for DSM IV [97], and a psychiatric consultation, non-smoking and had greater than 17 on the 17 item HDRS. **The MAO-A DV_S is highly significantly elevated ($p<0.001$ each region, average magnitude 34 per cent (or two standard deviations)) in the depressed subjects.**

The data from the 2006 is the definitive work to show that MAO-A is elevated in early onset depression because the magnitude is large, the sample is carefully defined, the method is selective for MAO-A and there has never been a post mortem study of early onset depression in medication free subjects. Previous post mortem studies of MAO-A did not examine the question as to whether MAO-A is elevated in medication free, early onset depression: The most common reasons are lack of specificity for MAO-A, diagnostic non-specificity by sampling of suicide victims rather than depressed suicide victims, inclusion of subjects who recently took medication, and/or overdosed [98-102], no differentiation between early onset depression and late onset depression [98-102], and small sample size.

Subsequent to the study of 2006, there have been several replications of elevated brain MAO-A levels during major depressive episodes. We replicated our finding in a separate sample in 2009 [22](again in the Archives of General Psychiatry), and in 2011 a separate laboratory reported similarly elevated MAO-A levels and activity in a postmortem study of the prefrontal cortex (the region sampled)[103]. Beyond this data, several recent studies have also generated new evidence in support of elevated MAO-A levels during MDE: Investigations of nuclear transcription factors that regulate MAO-A levels in postmortem investigations of MDE sampling the prefrontal cortex find reduced mRNA and levels of MAO-A inhibitory nuclear transcription factor R1 and greater levels stimulating nuclear transcription factor TIEG2 [104-106]. A report of greater whole brain serotonin turnover during MDE [107] is consistent with greater MAO-A level since MAO-A density is highly correlated with MAO-A activity in brain [66-68] and MAO-A is the primary route of metabolism of serotonin in brain[80, 108].

2.5. A New Biological Model for Postpartum Blues

Since postpartum blues at a high severity increases risk for PPD [7, 9, 40, 41], countering the biological changes in postpartum blues may be useful so as to prevent both. A new model of postpartum blues is outlined below:

Part 1: Estradiol and estriol are produced by the placenta during pregnancy and plasma levels rise 100 fold and 1000 fold respectively [109]. After delivery, these levels drop abruptly with most of the decline occurring in the first three days with a modest decline thereafter [6, 7, 12].

Part 2: There is an inverse relationship between changes in estrogen and changes in monoamine oxidase A (MAO-A) density, synthesis and activity in cell lines, in regions of high MAO-A density in rodents (amygdala, cortex) , and regions of high MAO-A density in Macaque monkeys (dorsal raphe nucleus) [13-19]. **In humans, we found that MAO-A binding is elevated during days 4 to 7 postpartum** using [^{11}C]

harmine positron emission tomography (PET) (see figure 1 below). MAO-A is an enzyme that metabolizes serotonin, norepinephrine and dopamine in the brain [64]. [^{11}C] harmine PET is a method to measure an index of MAO-A density in living brain [21, 96, 110, 111].

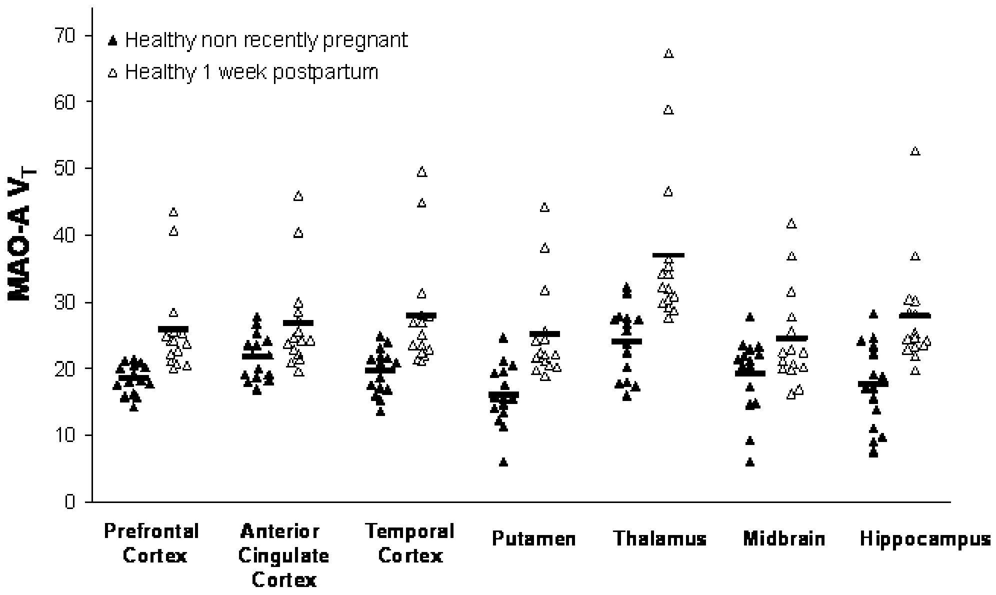

Figure 1: monoamine Oxidase A binding in the immediate postpartum period

Part 3: Greater MAO-A is expected to enhance metabolism of monoamines such as serotonin, norepinephrine and dopamine. Lowered monoamines (serotonin, norepinephrine and dopamine) leads to lower mood [24, 112-120].

There is quite a history of lowered monoamines leading to lowered mood: For example, reducing brain serotonin in humans through tryptophan depletion (reduction of substrate) lowers mood in humans, particularly when a family history of mood disorder or a past history of mood disorder is present[112-116]. Reducing dopamine and norepinephrine synthesis through administration of alphamethylparatyrosine lowers mood[117, 118]. Lowering all three major monoamines through administration leads to low mood in otherwise healthy individuals[24, 119, 120]. See **figure 2** below for an overview diagram.

An additional point is that heightened monoamine metabolism via MAO-A creates hydrogen peroxide, and markers of oxidative stress are associated with mood disorders[121, 122].

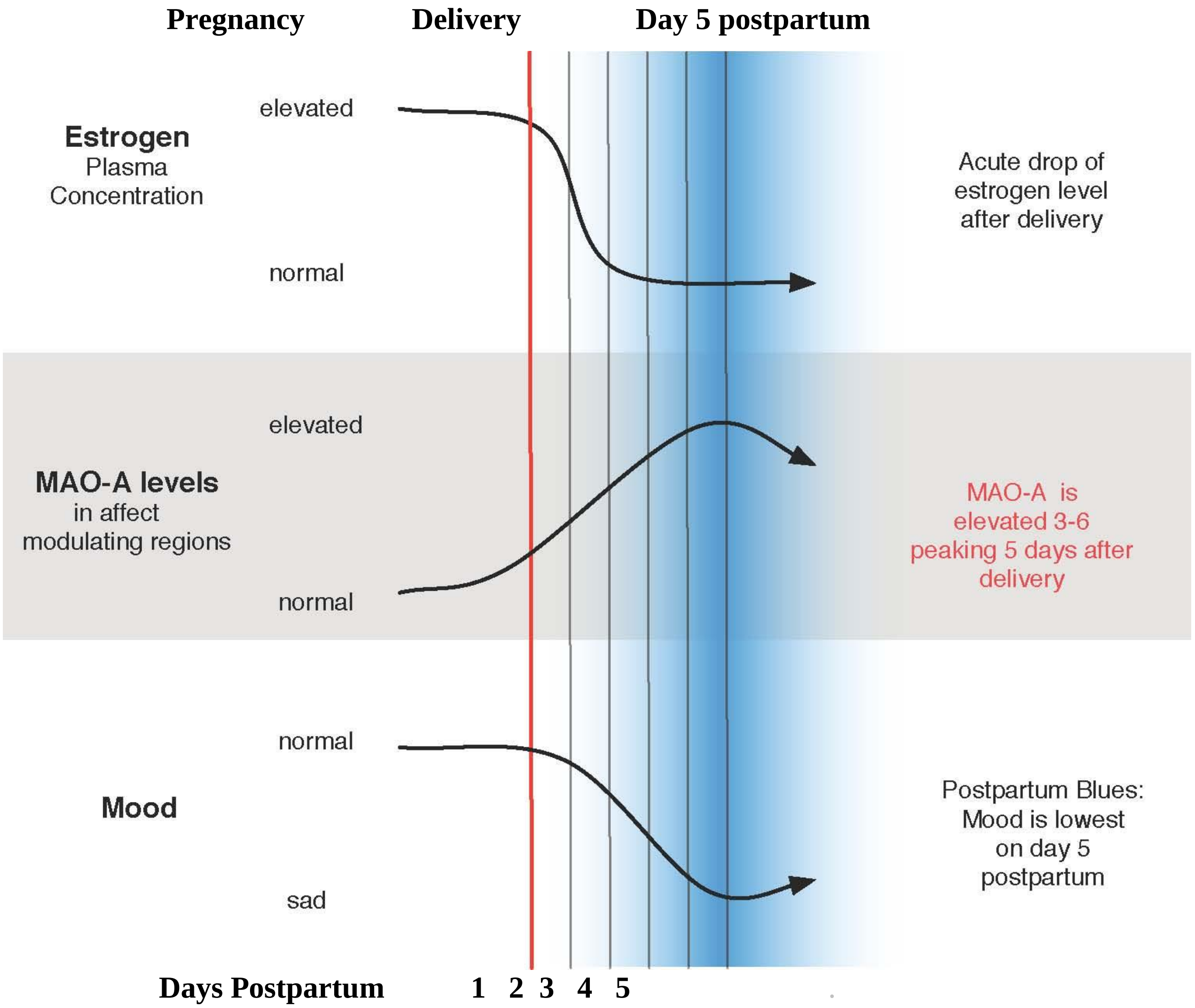

Figure 2. Monoamine theory of postpartum blues

2.6. Proposed Solution

As described in the previous section, consequent to the tremendous decrease in estrogens, MAO-A level is elevated in early postpartum throughout the brain, including the prefrontal and anterior cingulate cortex. We see this as a phenomenon that predisposes to postpartum blues and PPD.

The proposed solution is to counter the effects of high MAO-A levels by providing supplementation of amino acids which become serotonin, norepinephrine and dopamine (to replace monoamines) as well as blueberry juice with blueberry extract (for antioxidant effect). It is known that none of these neurochemicals directly cross the blood brain barrier, rather, precursor amino acids cross the blood brain barrier through a transport mechanism[123]. Tryptophan is a precursor amino acid for serotonin and tyrosine is a precursor amino acid for norepinephrine and dopamine[123, 124]. Both of these amino acids are transported via a carrier process that has a preference for large neutral amino acids[125]. In the brain,

tryptophan is converted by tryptophan hydroxylase into 5-hydroxytryptophan and then into serotonin via amino acid decarboxylase[126, 127]. In the brain, tyrosine is hydroxylated by tyrosine hydroxylase into DOPA which is then converted into dopamine by the enzyme dopa decarboxylase[126]. In neurons, that store and release norepinephrine, this neurotransmitter is formed by betahydroxylation of dopamine[126]. While many foods and fruits contain antioxidants[128], blueberries are chosen for their antioxidant content because they contain a number of different anthocyanins in contrast to other fruits which have mainly cyanidin anthocyanins[129]. A number of the anthocyanins, especially, malvindin and cyaniding anthocyanins have been reported to be detectable in the brains of blueberry fed rodents and pigs, and blueberry administration is associated with resistance to cognitive decline in rodents, suggesting again that the contents are brain penetrant[29-32, 130].

2.7. Advantages of Amino Acid Supplementation for Safety in Breast Milk

Many amino acids and most medications freely cross into breast milk. In contrast to medications, such crossing should not affect total concentrations of amino acids such as tryptophan and tyrosine into breast milk. The reason is that approximately 98% of tryptophan in breast milk is contained in proteins and peptides and that 99% of tyrosine in breast milk is contained in proteins and peptides[131-134]. This issue had never been investigated before. We have recently completed two separate studies investigating different doses of oral tyrosine (0 (none), 2, 5 or 10 grams) (n=6 each) or oral tryptophan (0g (none), 2 or 4 grams) (n=6 each) on their levels in breast milk. There was no change in total tryptophan levels in breast milk **(Figure 3)**, despite changes in plasma tryptophan levels **(Figure 4)**. Similarly after oral dosing of tyrosine, there was no change in total tyrosine levels in breast milk **(Figure 5)**, despite changes in plasma tyrosine levels **(Figure 6).** The amount of free tyrosine and free tryptophan in breast milk did increase, but since over 99% of these two amino acids are contained within proteins of fixed chains, thus their total levels did not change in breast milk after oral supplementation. We also examined this small percentage of free amino acid in breast milk and found that the levels of amino acid after oral supplementation were similar to levels found in many infant formulas **(Figures 7 and 8)**[34, 35].

Procedures that experimentally can change emotional state have been used for research and clinical reasons. Mood induction has been a cornerstone of research on experimental psychopathology and psychology [135-139].

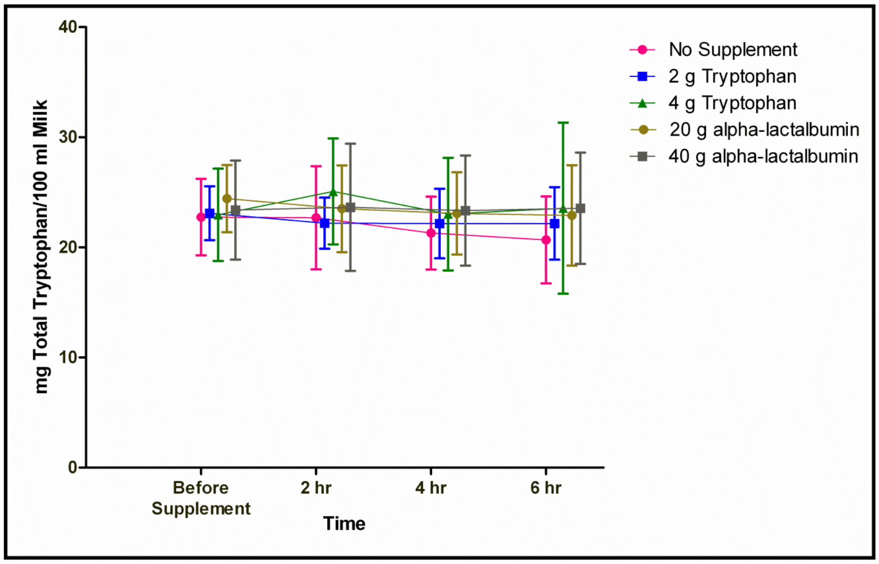

Figure 3. Total Tryptophan Concentrations in Breast Milk after Oral Tryptophan or Alpha-lactalbumin (a-lac) Supplements. There was no effect of group on repeated measurement of total tryptophan levels in breast milk (F (12,75)=1.31, p=0.232). Timing was identical for all groups. The data has been staggered for easier viewing. Error bars represent standard deviation.

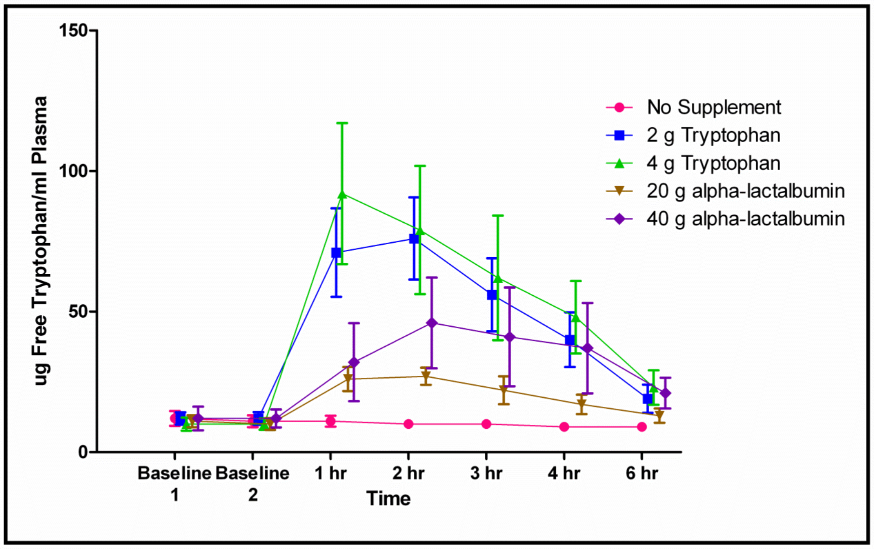

Figure 4. Plasma Free Tryptophan Concentrations after Oral Tryptophan or Alpha-lactalbumin (a-lac) Supplements. There was a significant effect of group on plasma levels of free tryptophan (F (24,150)=15.62, p<0.001). Timing was identical for all groups. The data has been staggered for easier viewing. Error bars represent standard deviation.

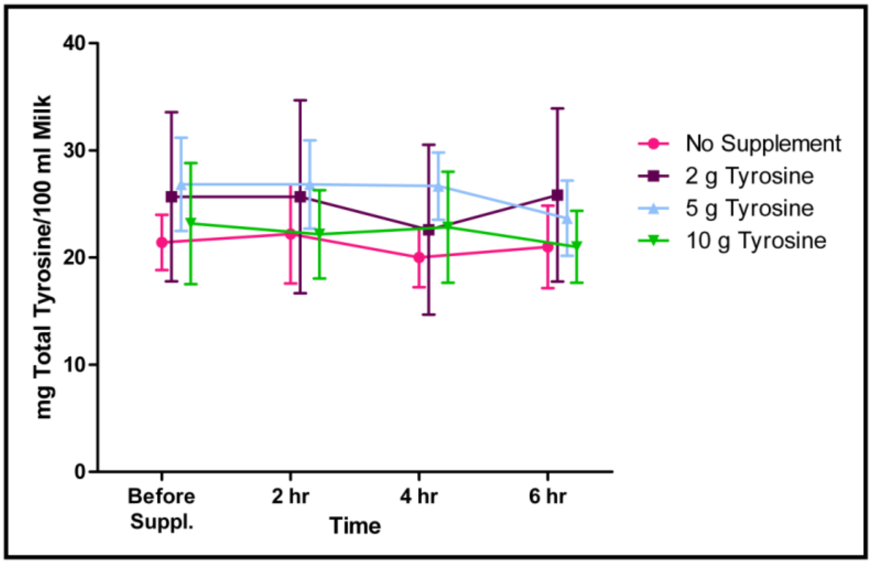

Figure 5. No Rise in Total Tyrosine Levels in Breast Milk After Oral Tyrosine Supplements. Repeated measures analysis of variance found no effect of group on repeated measurement of total tyrosine levels (F (3,20)=1.24, p=0.321). Timing was identical for all groups. The data has been staggered for easier viewing. Error bars represent standard deviation.

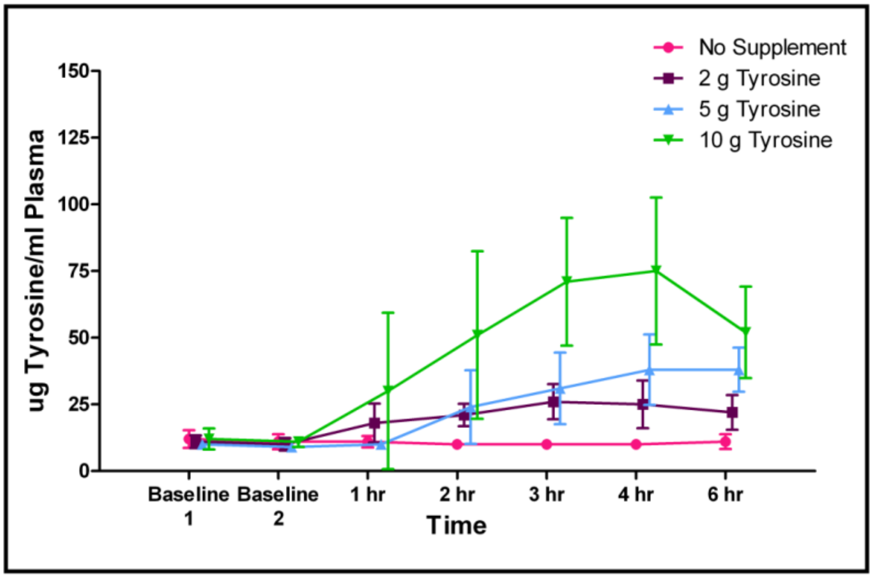

Figure 6. Elevation in Plasma Tyrosine Levels Over Time After Oral Tyrosine Supplements. Repeated measures analysis of variance showed a strong effect of group on plasma levels of free tyrosine (F (3,20)=33.67, p<0.005). Timing was identical for all groups. The data has been staggered for easier viewing. Error bars represent standard deviation.

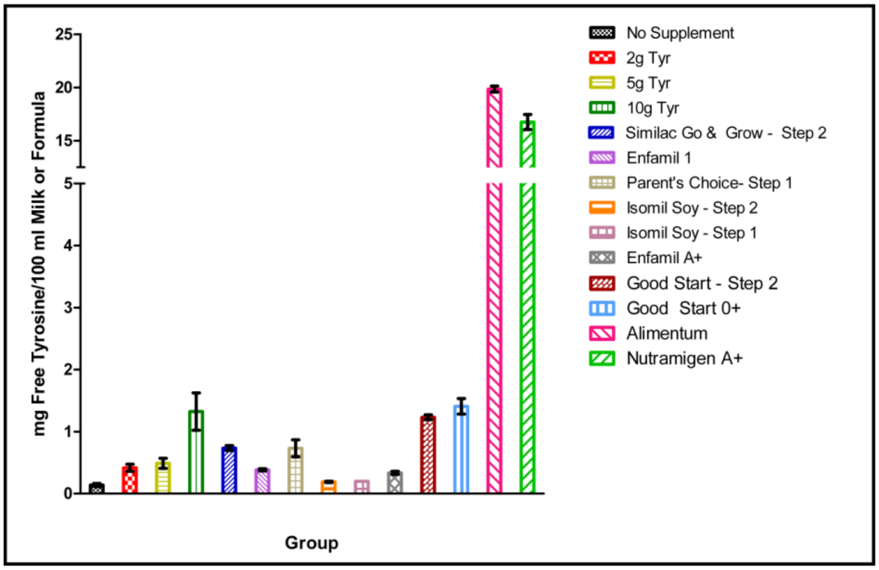

Figure 7. Free Tyrosine in Common Infant Formulas and in Breast Milk After Oral Tyrosine Supplementation. Mean and standard deviation are presented. Free tyrosine in breast milk even with the highest dose of 10g oral tyrosine was significantly lower than free tyrosine in the 2 extensively hydrolyzed infant formulas tested (Alimentum and Nutramigen A+) (Analysis of variance, post-hoc comparisons, Fisher's least significant difference (LSD), $p<0.005$) and similar to partially hydrolyzed formulas tested (Good Start 0+ and Good Start 2).

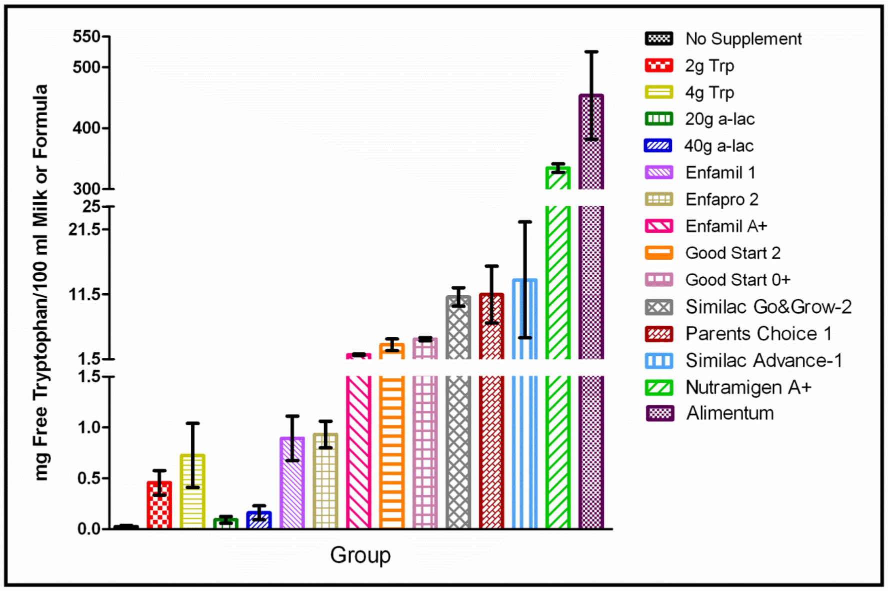

Figure 8. Free Tryptophan in Common Infant Formulas and in Breast Milk after Oral Tryptophan or Alpha-lactalbumin (a-lac) Supplementation. To capture the variation in free breast milk levels, the y axis was broken to 3 segments. Mean and standard deviation are presented. Free tryptophan in breast milk even with the highest dose of 4g tryptophan was comparable to non-hydrolyzed formulas, and significantly lower than extensively hydrolyzed formulas (Nutramigen A+ and Alimentum) (analysis of variance, post-hoc comparisons, Fisher's least significant difference (LSD), $p<0.005$)

2.8. The Effect of the Proposed Dietary Supplement on Mood Symptoms in Early Postpartum in an Open Label Trial

Rationale for Amounts Tested: Based on the optimal doses found in the studies investigating the effect of oral tryptophan and tyrosine on their concentrations in breast milk, we continued on investigating the ability of a dietary supplement containing 2 grams of tryptophan, 10 grams of tyrosine along with blueberry extract/juice as an antioxidant on reducing the intensity of postpartum blues. Tyrosine supplement at the dose of 10 grams was chosen to be tested since based on our previous clinical trial this dose did not have any effects on total tyrosine levels in breast milk, was significantly increased in maternal plasma and was extremely well tolerated. Moreover, most of the investigations of tyrosine with similar doses support its ability to protect against adverse cognitive effects of stress: Under conditions of cold stress, noise stress, and hypoxic stress, single doses of 100mg/kg [140, 141], 150mg/kg [142-145]and 300mg/kg [146, 147] were helpful in creating resilience, based upon unimpaired performance upon cognitive tasks, mainly in the area of working memory and again these doses were also well tolerated. Tryptophan supplement at the dose of 2 grams was chosen to be tested since based on our previous clinical trial this dose did not have any effects on total tryptophan levels in breast milk, was significantly increased in maternal plasma and was extremely well tolerated. Although 4 grams did not increase total tryptophan in breast milk, it was associated with nausea in half of the subjects receiving that dose. Moreover, the changes in plasma tryptophan between the groups receiving 2g or 4g were minimal, suggesting limited advantage of the greater dose. In a study administration of 0.8 grams of tryptophan, tryptophan was able to modestly, but significantly, improve mood in healthy subjects [148]. We acknowledge that it has been proposed that higher doses of tryptophan are necessary to reduce symptoms of major depressive episodes, although, . Hence, 2g, while lower than the 4g dose previously tested, may achieve at least modest benefit for mood as it is well tolerated, has no effects on overall tryptophan in breast milk and may have effects upon mood in healthy people.

Rationale for Postpartum Blues Target and Recent Advances in Quantitation of Postpartum Blues: We chose postpartum blues because when postpartum blues are more severe, they are associated with greater risk of postpartum depression (so reducing the intensity of postpartum blues should lead to reduced risk of PPD) [7, 41, 149]. We have also improved upon existing methods to measure the severity of postpartum blues through use of depressed mood induction and standardized mood rating scales after the mood induction, which is a standardized sensitive approach for measuring mood dysregulation[37, 38, 139]. We find that after sad mood induction, women who are day 5 postpartum and healthy women within the first 18 months postpartum with vulnerability to crying (but did not have clinical depression) clearly have greater shift towards depressed mood on visual analogue scale **(figure 9)** and the profile of mood states as compared to healthy women who are not in day 5 postpartum and do not have crying spells with in the first 18 months postpartum respectively. We have advanced symptom targeting through improving upon existing methods to measure the severity of postpartum blues[36]: We apply depressed mood induction and standardized mood rating scales after the mood induction, which is a standardized sensitive approach for measuring mood dysregulation, particularly in early postpartum[37, 38, 139].

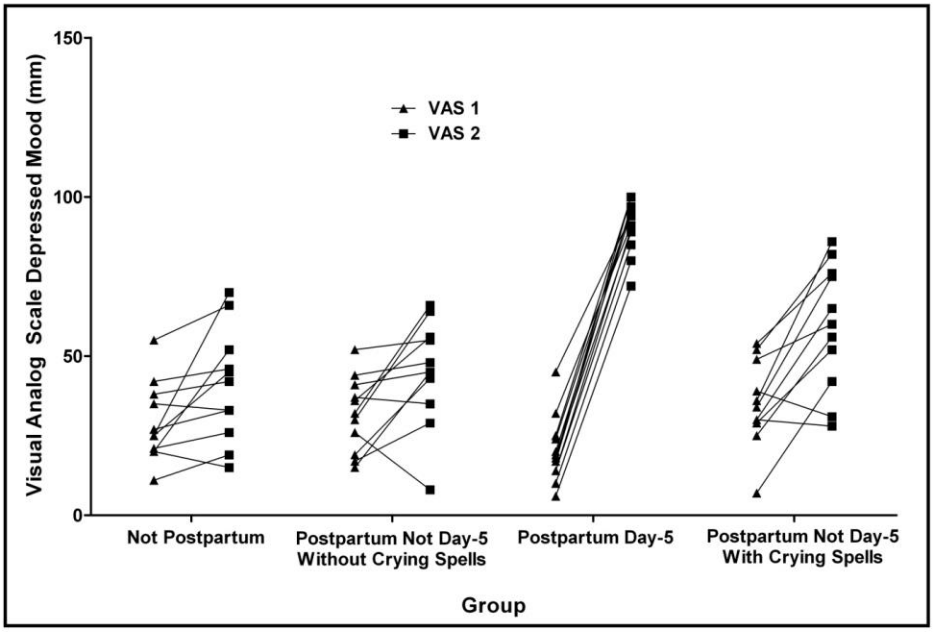

Figure 9. Stronger Elevation in Depressed Mood Scores in Visual Analog Scale (VAS) in Day-5 Postpartum Women. Univariate analysis of covariance showed a significant group effect on the change in VAS mood scores after the sad mood induction procedure ($F(3,41)=22.45$, $p<0.005$)

Results of the Previous Open Trial: We find that after sad mood induction, women who are day 5 postpartum, not taking the proposed dietary supplement, clearly have greater shift towards depressed mood on visual analogue scales **(figure 10)** and the profile of mood states as compared to healthy women who are in day 5 postpartum taking the proposed dietary supplement. The results of the open label trial, which is nearing completion, indicate that the dietary supplement was able to significantly attenuate the intensity of postpartum blues in day-5 postpartum women compare to those not receiving any dietary supplement. The results demonstrated that receiving the dietary supplement was significantly associated with minimal or no change in mood as measured by the VAS mood scores after sad mood induction. VAS depressed mood score change after the sad MIP was 50.03±16.29 in controls and 1.37±8.47 in supplemented group. The effect size was 5.4.

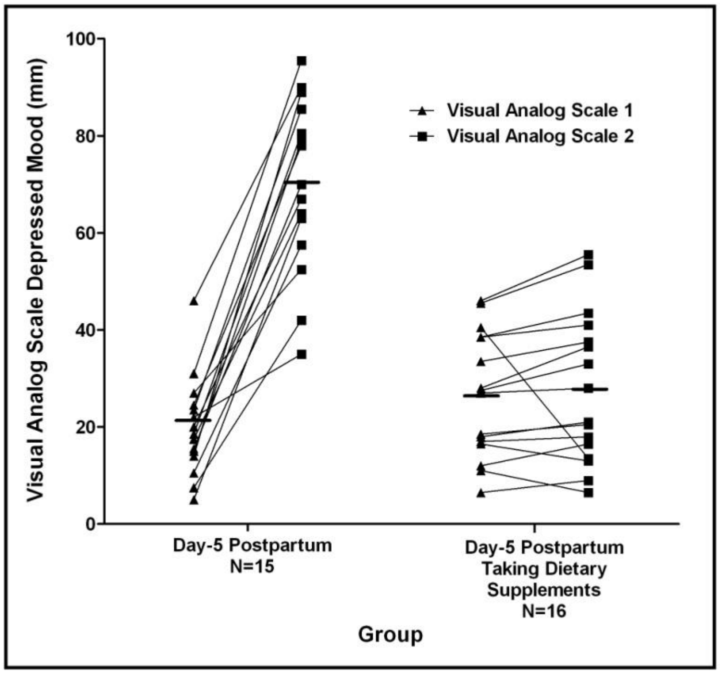

Figure 10. Stronger Elevation in Depressed Mood Scores in Visual Analog Scale (VAS) in Day-5 Postpartum Women not Taking the Dietary Supplement. Analysis of variance found significant elevation in depressed mood scores as measured by VAS in day-5 postpartum women not taking any supplements compared to day-5 postpartum women taking the dietary supplement ($F(1,29)= 110.91$, $p<0.001$).

Why advance from an open trial to a randomized double blind placebo controlled trial? Now in this proposed study we would like to investigate if a dietary supplement given in double blind placebo controlled RCT study can prevent the shift in sad mood in these two groups. The reasons to peruse investigating the effect of the proposed dietary supplement in a double blind placebo controlled trial include: 1) Significant positive effect of 5.4 observed in the recent open-label trial study, 2) The proposed dietary supplement has been well tolerated by all subjects and no side effects were observed, 3) We have an available placebo which was developed and tested in an ongoing separate clinical trial in our group, 4) A double blind randomized placebo controlled trial is expected in science. 5) If the combination of ingredients is someday developed as a dietary supplement, it is expected that a rigorous scientific process such as a randomized double blind placebo controlled trial is needed to demonstrate efficacy.

3. Hypothesis:

Sadness after depressed mood induction during postpartum blues will be attenuated in those receiving the dietary supplement (tryptophan, tyrosine and blueberry juice/extract) compared to placebo.

4. Objectives:

Primary endpoint:

To assess the ability of the dietary supplement to reduce the intensity of postpartum blues in a double blind RCT.

Secondary endpoint:

To assess the ability of the dietary supplement to prevent postpartum depression up to 6 months postpartum

5. Methods:

5.1. Subject criteria

One hundred forty pregnant women who are healthy and have no history of MDE will be recruited during their third trimester and such that at least 100 completing subjects will be enrolled to complete the study. Participants will be recruited by advertisement (see end of section 5.1 for further detail regarding advertising). Subjects expressing interest at earlier times in pregnancy will have their initial visit booked in the third trimester (i.e. in person completion of screening questionnaires to rule out a psychiatric disorder and final verification of other requirements). Subjects will randomly be assigned to placebo or active supplement in a double blind manner within blocks of 2, 4, 6, 8 or 10 subjects. Block size will be randomly selected using standard randomizing software. The supplements/placebo will be taken at 4 time points, starting on day 3 postpartum night to day 5 postpartum morning (See table 2 for full schedule). Subjects will receive either the dietary supplement or a placebo that matches in taste and colour (as verified by independent manufacturer and investigator for visual and taste testing during placebo product development) (this has already been created). On day 5 postpartum, participants will be seen for a single visit (at their house).

Inclusion Criteria:

I. The subject, as reported by them, should be in good health.

II. The subject is not taking any medication, with the exception of medications used to manage illnesses or conditions not implicated in affecting mood state.

III. The subject is not taking any investigational medicinal product within 8 weeks.

IV. Age 18 to 45

V. BMI 18.5 to 40 (kg/m^2) (based on participant self-report of pre-pregnancy weight and height)

VI. Resting pulse between 45 and 100 bpm

VII. Systolic blood pressure between 91 and 139 mmHg (inclusive)

VIII. Diastolic blood pressure between 51 and 90 mmHg (inclusive)

IX. Orthostatic blood pressure change <20 mmHg (based on the difference between supine and standing (1 minute) systolic blood pressure)

Exclusion Criteria:

I. The subject has been diagnosed with any disorders based on Structured Clinical Interview (SCID) for DSM-5 interview [150] within the past 10 years.

II. Subjects who have been smoking cigarettes in the past 5 years (to reduce variability in mood attributable to cigarette withdrawal and MAO-A).

III. Current substance abuse

IV. Known intolerance to specific components of tryptophan, tyrosine and blueberry

V. Currently taking MAO inhibitors and serotonergic drugs (including serotonin reuptake inhibitors; serotonin and norepinephrine reuptake inhibitors; tricyclic antidepressants; triptans; lithium; dextromethorphan; opiate medications with off target serotonergic effects such as methadone, pethidine (merperidine), tramadol, fentanyl, tapentadol; and serotonin agonists), as well as antiparkinsonian drugs. Ondansetron, an anti-nausea agent which is a blocker of serotonin at the 5-HT3 receptor and opposes effects of serotonin is not included as exclusionary

VI. Currently taking morphine which would affect mood

VII. Any medical illness that would affect mood state as assessed by the principal investigator

Exclusionary Medical Illnesses include:

1) Endocrine diseases requiring ongoing medication including hypo or hyperthyroidism
2) Frequent migraines (greater than one per week on average)
3) Neurological illnesses with the exception of infrequent migraines, and benign essential tremor
4) Neoplasm that is not benign
5) Pre-eclampsia/eclampsia
6) Autoimmune diseases without full recovery for more than 5 years
7) Metabolic diseases

Withdrawal criteria:

Participants who have been enrolled into the study as per the eligibility criteria listed above would be withdrawn from the study, should they meet any of the following criteria:

I. If in the principal investigator's judgement the obstetric or neonatal complications were so severe that would cause change in the results of the study, such as infant death, the subject will be withdrawn from the study (the purpose is to avoid undue burden on participants and to avoid biased results of mood induction).

II. Evidence of substance abuse based on the urine test results done on day-5 postpartum (the results of the urine test would be available 1-2 weeks later but removal of mood induction data biased by substance abuse would improve data integrity).

III. If the participant shows any allergic reaction or significant side effects to any components of the dietary supplement that would make continued participation harmful.

IV. Participant is recommended or required by their health care practitioner to take methadone, pethidine (merperidine), tramadol, fentanyl tapentadol, or morphine within 12 hours prior to taking their tryptophan dose scheduled on day 4 postpartum.

V. If in the principal investigator's judgment continuing the study is no longer in the participant's best interest.

Advertising

Study advertisements, flyers and brochures will be used for recruiting participants. This includes the following:

- Use of the CAMH Research Registry, CAMH member database and the CAMH website such as camh.net
- Advertisements/flyers/brochures posted in the community (including community centres, university campuses, natural health food stores, drugstores, exercise centres, community boards, public transportation, religious centres, coffee shops, health organizations, and maternal health and wellness organizations)
- Advertisements/flyers in online and print magazines, newsletters, and newspapers
- Advertisements/flyers/brochures and presentations at relevant conventions/shows
- Online/website advertisements on social media including those of health organizations, and maternal health and wellness organizations (including their Facebook, Twitter, Instagram, and blog accounts); websites Kijiji and Craigslist; and search engines (like Google and Yahoo)
- Advertisements/flyers/brochures posted in clinics of doctors and midwives
- Advertisements/flyers/brochures posted in hospitals

5.2. Dietary Supplements

The active dietary supplement consists of: 2 grams of tryptophan, 10 grams of tyrosine, blueberry juice and blueberry extract (with different schedules of administration for each). The tryptophan will be 1 g tablets (exactly what has been tested in our previous studies). Tryptophan will be obtained as L-tryptophan tablets from Apotex. Each container contains 100 of 1 gram tryptophan tablets. Tyrosine will be obtained as L-Tyrosine capsules from Natural Factors. Each container has 90 capsules of 500 milligram L-tyrosine capsules. The blueberry extract powder is called VitaBlue and will be purchased from Future-Ceuticals. The active blueberry pouches contain 2.02 grams of VitaBlue (product N1077) and 11.54 grams of sugar from Caldic. These pouches will be packaged by NSF-GFTC. The blueberry juice consists of 35.5 grams of blueberry juice concentrate from ProIngredients MILNE, 2.66 grams of blueberry flavor from Bell Flavors, 21.3 grams sugar and 0.36 grams of citric acid from Caldic and 282.63 grams of water. The blueberry juice is packaged in glass containers by NSF-GFTC. With the exception of the tryptophan and tyrosine, the amounts listed for each item listed in the active dietary supplement and placebo (see below for placebo), are approximate, being within 5% of the listed amounts.

The placebo consists of: The placebo pills appear identical to tryptophan and tyrosine pills and will be prepared by CAMH pharmacy. Empty hard gelatin capsules from Capsugel and Lactose monohydrate from Galenova for placebos to match tryptophan and tyrosine. Tryptophan pills will also be over encapsulated to match the placebos. The placebo pouches (to match the blueberry extract) contain 1.02 grams of Shade grape blue powder from GNT and 12.54 grams of sugar from Caldic. The placebo drink is a natural drink that is blue through natural colouring, has a flavour like blueberry through natural flavoring, and has negligible antioxidant properties and similar sugar levels to the active drink. The placebo blueberry beverage consists of 3.55 grams of blueberry flavor from Bell Flavors, 21.30 grams of sugar and 0.71 grams of citric acid from Caldic, 0.89 grams of Shade Bordeaux from GNT and 316 grams of water. The placebo pouches and placebo beverages are package by NSF-GFTC.

Timing of administration of study products:

- Night of day 3 postpartum: Participants will mix one pouch of active or placebo extract with one active or placebo juice and drink at once
- Morning of day 4 postpartum: Participants will mix one pouch of active or placebo extract with one active or placebo juice and drink at once
- Night of day 4 postpartum: Participants will mix one pouch of active or placebo extract with one active or placebo juice and take the 2 grams of tryptophan (2 capsules of 1 gram tryptophan each) or placebo with the beverage
- Morning of day 5 postpartum (main study day): Participants will mix one pouch of active or placebo extract with one active or placebo juice and take the 10 grams of tyrosine (20 capsules of 500 milligrams tyrosine each) or placebo with the beverage

Blinding: The ratio of active treatment to placebo will be 1:1. Since the supplement appears likely to prevent postpartum blues on an individual case level, additional care is being taken in regards to the randomization process. The assignment of placebo supplement or active supplement will be done by the research pharmacy department who randomizes subjects evenly to each condition within blocks of 2, 4, 6, 8 or 10 subjects. Each block size will be randomly selected using standard randomizing computer software. Then, for each block subjects will be randomly selected to either protocol. For example, if a block is of size 8, four ordered numbers will be randomly assigned to one group and the remaining 4 numbers will be assigned to the other group. Each subject will be recorded in relation to a code number. The administration of the mood induction procedure and the supplement will be done on day 5 postpartum by an expert who is blinded to both.

5.3 Study Overview

The study will involve a minimum of 9 phone calls, the first including a pre-screening questionnaire, and 2 in person visits (screening and day 5postpartum visit). See Table 1 for a description of the order.

A Dietary Supplement for Mood Symptoms in Early Postpartum: A Double Blind Randomized Placebo Controlled Trial

Table 1. Schedule of Events

Procedure	Pre-Screen Phone	Phone Call/ Webex	Phone Call	Visit 1 In Person	Enrollment (confirm eligibility)	Phone Call (Supplement delivery)	Phone Call (2 wks prior to EDD)	Phone Call (repeat if need)	Day 3 PP (Phone)	Day 4 PP (Phone)	Day 5 PP Visit 2 In Person	10 day PP	1 Mo. PP	3 Mo. PP	6 Mo. PP
Pre-screening Questionnaire	X														
Consent Review		X													
Sign Consent				X											
Inclusion/Exclusion Criteria				X	X										
Screening Questionnaires[a]				X											
Medical History				X	X										
Urine Test				X							X				
Confirm Birth							X								
EPDS & SCID DSM-5 questionnaires							X								
Delivery Status								X							
Review Concomitant Med.				X				X	X	X	X				
Instructions on Supplement Use						X		X	X	X					
Mood Induction											X				
Day 5 PP Questionnaires[b]											X				
Compliance Verification											X				
Order Supplement					X										
Adverse Events									X[d]	X[d]	X[d]				
Follow-up Symptom Questionnaires[c]												X	X	X	X
COVID-19 Screening			X[e]						X	X					

a: Screening questionnaires done on visit 1 include: SCID-RV for DSM-5, BDI, HAM-D, EPDS, DAS, SCID-5-SPQ, NEO PI-R, Believability Scale
b: Day 5 PP (Postpartum) questionnaires include: PSQI, VAS, POMS, DAS A and B, STAI, Emotional Stroop, EPDS, BDI, HAM-D, Kennerley Gath, CES-D, Steins Blue
c: Follow-up symptoms questionnaires include: SCID-RV (for mood, anxiety and OCD), EPDS, BDI, HAM-D, CES-D, Kennerely Gath, Steins Blue
d: This will be through Safety and Tolerability Questionnaire. The day 3 PP safety and tolerability measurements are collected as baseline symptoms prior to IP intake.
e: If "yes" to screening questions prior to first visit, this may be repeated 4 weeks +/- 4 days later if time is available to complete all study procedures.

5.4 Study Procedures

5.4.1. First Phone Call (pre-screening)

Prior to scheduling participants for their first screening visit, they will undergo a pre-screening questionnaire over the phone, which covers questions related to the likelihood of the participant being eligible for the study. The questionnaire involves pregnancy status, substance use information, medical history and psychiatric history. At the end of the questionnaire, participants will be scheduled for their screening visit.

The day before the first visit, the participant will be contacted by phone and asked COVID-19 screening questions. If a participant has been screened as positive (answered "yes" to any of the questions), she will be rescheduled for the same phone call to ask about COVID-19 screening questions as well as the first visit, 4 weeks +/- 4 days later, provided there is sufficient time then to complete the remaining study procedures. If there is again a "yes" answer to the COVID-19 screening questions, then the participant will not continue in the study.

A copy of the consent form will be sent to the participants (in electronic email format) prior to their first visit. This will allow the participant to read about the study and ask questions prior to the first visit.

5.4.2. Optional Initial Review of the Informed Consent Form (Via Webex or Phone Call)

Participants will be offered an optional remote consent discussion to review the Informed Consent Form with a study staff member prior to in-person consent procedures. This webex appointment is greatly preferred because it increases the amount of time presenting the consent form to study participants, however, some study participants may not wish to have the webex appointment, so it is optional. After participants have received a copy of the Informed Consent Form via email, a time will be arranged (by webex or phone call) in order to discuss and review the Informed Consent Form. During this conversation, all questions raised will be addressed or followed up.

5.4.3. First Visit

The first in-person visit will start by completing the consent process, following up on any questions and addressing all questions raised by the study participant and then, should the participant consent, screening processes follow. This visit will take place at the participant's residence (During all the in-person visits, regional and provincial guidelines for COVID prevention, such as use of personal protective equipment (PPE), will be followed for the safety of both study participants and research personnel). On this day subjects will be evaluated for general health status through a standardized health questionnaire. Height and weight will be collected to evaluate body mass index (BMI), as measured in either imperial or metric units, but not a mix of both. Resting and orthostatic blood pressure, and heart rate measurements will be taken. SCID DSM-5 [150], Beck Depression Inventor Scale (BDI) [151], Dysfunctional Attitude Scale (DAS) [152, 153], The Revised NEO Personality Inventory (NEO PI-R) [154], Hamilton Rating Scale for Depression (HAM-D) [155], Edinburgh Postnatal Depression Scale (EPDS) [156] and the believability scale [157] are filled out. A record of the use of any vitamin, mineral, natural health product, herbal product and any medication will be obtained and recorded. Subjects will be asked about their education, ethnicity, socioeconomic, marital, and employment status. A urine test will be done in order to screen for any drug use in pregnancy.

This urine drug screen is done to screen participants who overlook reporting of their use of medication or substances and a positive result would be considered an indication of possible substance abuse disorder leading to exclusion from the study.

- SCID-5-RV and SCID-5-SPQ will be applied for each subject. This is a diagnostic exam used to determine DSM-5 major mental disorders and personality disorders [150].
- BDI is the most widely and validated self-report test, which measures the existence and severity of depression. It asks how one felt over the past seven days[151].
- EPDS is a 10-item questionnaire that was developed to identify women who have PPD. Items of the scale correspond to various clinical depression symptoms, such as guilt feeling, sleep disturbance, low energy, anhedonia, and suicidal ideation. It asks how one felt over the past seven days[156].
- HAM-D is a 21-item questionnaire used to provide an indication of depression[155].
- DAS is a 40-item instrument that is designed to identify and measure cognitive distortions, particularly distortions that may relate to or cause depression. Form A and form B of the DAS will be administered in a counterbalanced design between subjects[152, 153].
- NEO PI-R is recognized internationally as a gold standard for personality assessments and is a measure of the five major domains of personality including: Extraversion, Agreeableness, Conscientiousness, Neuroticism, and Openness to Experience; as well as the six facets that define each domain. This test is 240 items[154].
- Believability scale is a questionnaire designed to assess the subject's belief [157]. There are two proposed. The first is in regards as to whether they will receive the placebo or dietary supplement. It is a 10cm scale with the statement, "I think I will get the placebo" at one end and "I think I will get the dietary supplement" on the other. The second is a 10 cm scale reflecting their belief that the dietary supplement affects mood, "I think the dietary supplement being tested prevents depressed mood" is at one end and "I think the dietary supplement does not prevent depressed mood" is at the other end. The participant will put an X on the 10 cm scale. For scoring purposes, the rater will mark the midpoint of the X and measure the distance from the beginning of the scale on the left side with a ruler. At the end of the study (6 months postpartum), the first question will be repeated over the phone, but in the past tense, with the statements, "I think I received the placebo" on one end, and "I think I received the dietary supplement" on the other end[157]. The rater will ask the participant to report their view on a scale of 0 to 100.

5.4.4. Procedures after First Visit and Before Giving Birth

- Enrollment confirmed based on participant's full eligibility once urine test results have been obtained and assessed.
- Subjects will be randomized by CAMH pharmacy in a double-blind manner for receiving the dietary supplements (i.e. the rater and the subject will be blinded) (See section 5.2).
- Subjects will receive the package containing dietary supplement package for doses to be taken on day 3 postpartum night, day 4 postpartum morning, day 4 postpartum night and day 5 postpartum morning up to two months prior to their estimated due date. There will be a phone call to arrange a delivery time for the supplement and to provide instructions regarding its use.
- Participants will be contacted between 2 to 8 times, at a frequency of every several days to weeks based on their preference, and estimated time until due date in order to gain update about their delivery status. We will suggest 2 to 3 times as being a reasonable number and then accommodate participant preference.

- Two weeks (+/- 5 days) prior to the subjects estimated due date, they will receive a follow-up phone call in order to ask about their present mood and feeling and will involve completing the current mood questionnaires (current MDE, current manic episode, current cyclothymic disorder, current persistent depressive disorder) on SCID-RV for DSM-5, and EPDS.

5.4.5. Procedures after Giving Birth and Before Day 3 Postpartum

- Upon notification of child delivery, the participants will be asked about method of child delivery, presence of any neonatal or obstetrical complication during or after the delivery, if the baby was transferred in to any special care unit or not. They will also be asked if there were any changes to their concomitant medication. This information will be recorded. The principal investigator (PI) is then notified of the results. If in the principal investigator's judgement, the obstetric or neonatal complications were so severe that would cause change in the results of the study, such as infant death, the subject will be withdrawn from the study.
- Subjects will be notified about the day that the dietary supplement should be started and when their day 5 postpartum visit will be. For the purpose of identifying the day 5 postpartum visit, the following calculation will be followed:
    - Collect the delivery date and time.
    - If a participant has given birth before 6 pm, consider the day of delivery as 1 postpartum.
    - If birth time is after 6 pm, consider the day of delivery as day 0 postpartum and the next will be considered as day 1 postpartum.

5.4.6. Procedures from Day 3 to Day 5 Postpartum

- Subjects will start taking the dietary supplement from day 3 postpartum (as shown in Table 2 section 5.4). They will be ingesting a total of 4 drinks over the course of 3 days, which 3 of them will be taken over 2 days prior to the second visit (two drinks are mixture of blueberry juice and blueberry extract, and one will be 2 g tryptophan in the form of tablets taken with the mixture of blueberry juice and blueberry extract) and the fourth drink mixture will be taken on the second visit which will be 10 g tyrosine in the form of capsules taken with the mixture of blueberry juice and blueberry extract.
- Each participant is contacted 3 times to be reminded for dietary supplement intake.
    - On day 3 postpartum night prior to the intake of the first dose, they will receive a call between 6 to 9 pm. At least 3 tries should be made if unavailable at first call. The calls will be documented in contact log. Safety and tolerability checklist and concomitant medications review will also be completed on day 3 postpartum. During this phone call, the participant will also be asked about any symptoms of COVID-19. If a participant has been screened as positive (answered "yes" to any of the questions), she will be withdrawn from the study.
    - This procedure will be repeated on day 4 postpartum morning between 8 am to 10 am and prior to the intake of the second dose and,
    - One on day 4 postpartum night between 6 pm to 9 pm prior to the intake of the third dose. The safety and tolerability checklist and concomitant medications review will be completed on day 4 postpartum night. During this phone call, the participant will also be asked about any symptoms of COVID-19. If a participant has been screened as positive (answered "yes" to any of the questions), she will be withdrawn from the study.

The last dose will be taken in front of the study co-investigator or study RA on day 5 postpartum.

The second visit would be the main study day. The timing of each test done during this visit is shown in Table 2. On day 5, the starting time and subsequent time for the procedures may be delayed up to 6 hours in the event of childcare needs or doctor's appointments for the participant; or other similarly important appointments for the participant; or if the study team is unavailable due to multiple births on the same day.

- At the beginning of this visit, participants will have their concomitant medications reviewed and then take the fourth part of the IP. Resting blood pressure and heart rate measurements will be taken. They will do a urine test as well. This test will be considered as a withdrawal criteria. Compliance is also assessed (see more detailed description later in this section). They will also be asked about any neonatal or obstetrical complications.
- Afterwards, they will complete the following instruments:
    - Pittsburgh Sleep Quality Index, contains 9 self-report questions and assesses the sleep quality [158]. This questionnaire will be used to assess the sleep quality of the night before day-5 postpartum.
    - Subjects will go through a neutral mood induction based on the Velten[37] Mood Induction Procedure, which is the most widely used technique for studying affective influences upon behavior and it has demonstrated effectiveness in altering subjective emotional states (Frost & Green, 1982). To facilitate the neutral MIP, participants will also be presented with a piece of neutral music, from work by Clark et al [38] (See full detail below).
    - Following the neutral MIP, subjects will fill out the following questionnaires in the stated order:
        - Visual Analog Scale (VAS) uses a 10-point scale for participants to indicate the extent to which each of the 8 items is consistent with how they feel in the moment; the items included depressed, happy, restless, sad, anxious, angry, drowsy and alert[159]. Within VAS, mood assessment will be done first.
        - Profile of Mood State (POMS) contains of 65 adjectives rated by participants on a 5-point scale. Six factors are derived that include tension, depression, anger, fatigue, vigor and confusion[160].
        - DAS
        - VAS
        - State-Trait Anxiety Inventory consists of two scales containing 20 items each. One scale addresses state anxiety while the other scale addresses trait anxiety. The total score indicates which type of anxiety is prevalent and distinguishes between a person's state and trait anxiety levels[161].
    - Subjects will go through a sad mood induction, which will be done based on the Velten[37] Mood Induction Procedure, which is the most widely used technique for studying affective influences upon behavior and it has demonstrated effectiveness in altering subjective emotional states [162]. To facilitate the sad MIP, participants will also be presented with a piece of sad music, from work by Clark [38].
    - Following the sad MIP, subjects will repeat the following questionnaires in the stated order:
        - VAS
        - POMS
        - DAS
        - VAS

- After the completion of the above questionnaires, subjects will go through an Emotional Stroop Test. This test is used as an information-processing approach to assess emotions. The Emotional Stroop test works by examining the response time of the participant to name colors of negative emotional words.
- VAS
- Subjects will go through a neutral mood induction
- VAS
- BDI
- EPDS
- Stein Blue Scale is a self-rated scale and consists of 13 symptoms (depression, crying, anxiety, tension, restlessness, exhaustion, dreaming, appetite, headache, irritability, poor concentration, forgetfulness and confusion)[163].
- Kennerley and Gath is a self-rated 28 items blues questionnaire[164].
- HAM-D
- Center for Epidemiological Studies Depression Scale (CES-D)[165], is a 20-item depression inventory scored from 0 (least depressed) to 60 (most depressed).
- The second part of the Safety and tolerability checklist
- Record for diet on day 4 postpartum night and 5 postpartum morning

The second visit will take place at the participant's residence. As for all the in-person visits, appropriate personal protective equipment (PPE) and procedures will be used for the safety of both study participants and research personnel.

Compliance:

Participants will take 3 doses of the dietary supplement at home. They will receive a reminder phone call prior to each intake (see above). The fourth dose will be taken in front of the study administrator. To verify the compliance, for the first 3 doses the study administrator will ask the participant to show the 3 empty blueberry/placebo juice bottles, extract pouches and the empty tryptophan/placebo container. The empty bottles, empty pouches and empty tryptophan and tyrosine containers will be collected and returned to CAMH pharmacy.

Mood induction:

Sad mood induction will be done based on the Velten[37] Mood Induction Procedure, which is the most widely used technique for studying affective influences upon behavior and it has demonstrated effectiveness in altering subjective emotional states (Frost & Green, 1982). Velten mood induction procedure is a series of 60 self-referent statements. Negative statements reflected pessimism, dissatisfaction, and lethargy; for example "life is a heavy burden". Neutral statements examples are such as "an orange is a citrus fruit". Subjects will be asked to read each statement, printed individually, first to themselves and then aloud, and to 'feel and experience each statement as it would apply to you personally'. Subjects will be left alone in the room during the mood induction procedure. To facilitate the sad MIP, participants will also be presented with a piece of music, from work by Clark et al [38]. The music will be played simultaneously, while the subject is reading the statements. Subjects will listen to a piece of music that conveyed the tone of the mood trying to be induced. For sad MIP, subjects will listen to Prokofiev's Russia under the Mongolian Yoke' and for neutral MIP, Mozart's "Piano Concerto No. 21 in C Major"

will be played. Both are played at normal speed with a specific set volume. Subjects are asked to keep the same pace throughout the book.

Hence, overall, the process for depressed mood induction is to go through the Velten sad MIP, by reading 60 negative statements which is approximately 15-20 minutes long, accompanied by the listening of Prokofiev's Russia under the Mongolian Yoke done at normal speed. Then the assessment of depressed mood is done by completing the VAS, POMS, and DAS. The process of neutral MIP, is to go through the Velten neutral MIP, by reading 60 neutral statement, while listening to the Mozart's "Piano Concerto No. 21 in C Major", which also takes about 15 to 20 minutes.

Clinical rating measures of mood and symptoms of depression (i.e.: Visual Analog Scale and Profile of Mood States) will be assessed before and after MIP.

5.4.7. Procedures after Day 5 Postpartum

There will be four follow-up phone calls: Day-10 postpartum, 1 month postpartum, 3 months postpartum and 6 months postpartum. Follow-up phone calls will involve few general questions regarding participant's mood and feeling over the past few weeks, which involve completing the questionnaires SCID-RV for DSM-5 (mood (current MDE, current manic episode, current cyclothymic disorder, current persistent depressive disorder), anxiety disorders and OCD sections), HAM-D, EPDS, CES-D Stein blues Scale, Kennerley and Gath Blues Scale and BDI. Since history of COVID-19 may also affect mood state, participants will also be asked if they were diagnosed with COVID-19 at each phone call. Even though some of these questionnaires are self report, all of these questionnaires will be conducted over the phone by the study co-investigator, study RA, or research student. For each follow-up phone call at least 5 attempts will be done in the subsequent 10 days after scheduled time points (we do not leave messages). If the participant is unavailable, the 1 month postpartum, 3 month postpartum, and 6 month postpartum follow-up phone calls may be completed up to one week after the 10 day window.

Table 2. Subject's schedule during their second visit (main study day)

Day	*Time*	*Activity*
**Day 3 Postpartum Night**	9:00 pm to 10:00 pm	Blueberry Drink Intake (blueberry extract + blueberry juice) or Placebo<br>Safety and tolerability questionnaire
**Day 4 Postpartum Morning**	10:00 am to 11:00 am	Blueberry Drink Intake (blueberry extract + blueberry juice) or Placebo
**Day 4 Postpartum Night**	9:00 pm to 10:00 pm	Tryptophan & Blueberry Drink Intake (blueberry extract + blueberry juice) or Placebo<br>Safety and tolerability questionnaire
**Day-5 Postpartum (Main Study Day)** These procedures will take place in chronological order	8:30 am +/- 15 min	Meet with study participant
		Concomitant medications review
		Tyrosine & Blueberry Drink Intake (blueberry extract + blueberry juice) or Placebo
		Compliance assessment
		Urine Test (may be done any time in the morning)
		Pittsburgh Sleep Quality Index
	9:30 am +/-30 min	***Neutral mood induction procedure***
		VAS
		POMS
		DAS
		VAS
		State-Trait Anxiety Inventory
		Break
	10:20 am +/-30 min	***Sad mood induction procedure***
		VAS
		POMS
		DAS
		VAS
		Break
		Emotional Stroop Test
		VAS
		Break
	11:05 am +/-30 min	***Neutral mood induction procedure***
		VAS
		BDI
		Edinburgh Postnatal Scale
		Stein Blue Scale
		Kennerley and Gath Blue Scale
		Break
		HAM-D
		CES-D
		Safety and Tolerability Questionnaire
		Record of Diet

Abbreviations: MIP= Mood Induction Procedure, VAS= Visual Analog Scale, POMS= Profile of Mood State, DAS= Dysfunctional Attitude Scale, BDI= Beck Depression Inventory Scale

5.5 Statistical Analysis

Our primary analysis will be the use of repeated measures analysis of variance with the visual analogue scale of depressed mood as the repeated measure (before and after the sad MIP) and active condition versus placebo as the between subject measure.

Our secondary analysis will be the change in depression scores on profile of mood state (POMS) scale, using repeated measures analysis of variance with the POMS score as the repeated measure (before and after the sad MIP) and active condition versus placebo as the between subject measure.

5.6 Power

The mean change in VAS score after mood induction day 5 postpartum is approximately 45mm with a standard deviation of this difference of 12mm. To detect a reduction of change in VAS score shift such that the shift is 22.5mm rather than 45mm, reflecting a 50% reduction in the potency of the mood induction, with an alpha coefficient of 0.015, and a sample of 30 in each group, the power is over 95%. To detect a 25% reduction of change in VAS score with alpha coefficient of 0.015, sample of 30 in each group, the power is still 80% (power is done for 60 subjects, based upon a dropout rate of 10 subjects). With the POMS (profile of mood state scale), a similar analysis yields a power of 80% to detect a 25% reduction in the mood induced shift in POMS. We have proposed a trial of up to 100 completed subjects as this is a common standard in our field, although the power is very good for somewhat smaller numbers.

5.7 Safety and Tolerability Measurements

A safety and tolerability checklist will be filled out as a standard procedure for each subject on the phone on day 3 and 4 postpartum and in person on day 5 postpartum. The day 3 postpartum safety and tolerability measurements are collected as baseline symptoms prior to IP intake.

Blood pressure and heart rate will be taken at Visit 1 and on day 5 postpartum (Visit 2).

5.8 Drop Out/Early Termination

If participants drop out or have early termination from the study, they will be asked to return the supplement package (we offer to pick up the package at a time that is convenient for the participant). If this is not feasible for them, they will be asked to dump the blueberry/placebo juice bottles and return the tryptophan/placebo pills to a pharmacy to be discarded.

5.9 Adverse Events

Methods and timing for assessing, recording, and analyzing safety parameters:
Adverse events will be recorded on an Adverse Event Log. The Principal Investigator will be consulted to make a determination on the reasonable causal relationship to the investigational product and the seriousness of the event. Adverse events will be collected until the end of the Day 5 post-partum study visit.

Procedures for eliciting reports of and for recording and reporting adverse events:
Any Local Adverse Event that, in the opinion of the Principal Investigator, meets the definition of an unanticipated Problem will be reported to the CAMH REB according to the REB timelines as per their SOP. In addition, other incidents, experiences or outcomes that are not considered Adverse Events but, in the opinion of the Principal Investigator, meet the definition of an Unanticipated Problem, will be reported to the REB according to the REB timelines as per their SOP.

Every adverse event will be assessed and recorded in the subject's file by the Qualified Investigator or another delegated physician involved in the study.

The Principal Investigator will report all Serious and Unexpected Adverse Drug Reactions (SUADR) to Health Canada as per Division 5 regulations.

Type and duration of the follow-up of subjects after adverse event:
The Principal Investigator is responsible for determining the duration of follow-up required for any adverse event dependent on the seriousness of the event. Participants will be followed up until adverse event resolution or stabilization as determined by the Principal Investigator.

6.0 Additional Recording

The number of people to whom the study was explained on the phone or in person and their reason for disinterest/ineligibility will be recorded. The number of people who consented for treatment, and discontinued the study, and their reason for discontinuation will be recorded.

References

1. O'Hara MW, Swain AM. Rates and risk of postpartum depression: a meta analysis. *International Review of Psychiatry.* 1996;8:37-54.

2. Michaud CM, Murray CJ, Bloom BR. Burden of disease--implications for future research. *JAMA.* Feb 7 2001;285(5):535-539.

3. Grace SL, Evindar A, Stewart DE. The effect of postpartum depression on child cognitive development and behavior: a review and critical analysis of the literature. *Arch Womens Ment Health.* Nov 2003;6(4):263-274.

4. Robinson G, Stewart DE. Postpartum disorders. In: Stotland N, Stewart DE, eds. *Psychological aspects of women's health care*. Washington DC: American Psychiatric Press; 2001:117-139.

5. O'Hara M. *Postpartum depression: Causes and Consequences*. New York: Springer-Verlag; 1994.

6. O'Hara MW, Schlechte JA, Lewis DA, Varner MW. Controlled prospective study of postpartum mood disorders: psychological, environmental, and hormonal variables. *Journal of abnormal psychology.* Feb 1991;100(1):63-73.

7. O'Hara MW, Schlechte JA, Lewis DA, Wright EJ. Prospective study of postpartum blues. Biologic and psychosocial factors. *Archives of general psychiatry.* Sep 1991;48(9):801-806.

8. Romito P, Saurel-Cubizolles MJ, Lelong N. What makes new mothers unhappy: psychological distress one year after birth in Italy and France. *Social science & medicine (1982).* Dec 1999;49(12):1651-1661.

9. Brugha TS, Sharp HM, Cooper SA, et al. The Leicester 500 Project. Social support and the development of postnatal depressive symptoms, a prospective cohort survey. *Psychological medicine.* Jan 1998;28(1):63-79.

10. Wisner KL, Parry BL, Piontek CM. Clinical practice. Postpartum depression. *N Engl J Med.* Jul 18 2002;347(3):194-199.

11. Steiner M. Perinatal mood disorders: position paper. *Psychopharmacol Bull.* 1998;34(3):301-306.

12. Nott PN, Franklin M, Armitage C, Gelder MG. Hormonal changes and mood in the puerperium. *Br J Psychiatry.* Apr 1976;128:379-383.

13. Luine VN, McEwen BS. Effect of oestradiol on turnover of type A monoamine oxidase in brain. *Journal of neurochemistry.* Jun 1977;28(6):1221-1227.

14. Leung TK, Lai JC, Marr W, Lim L. The activities of the A and B forms of monoamine oxidase in liver, hypothalamus and cerebral cortex of the female rat: effects of administration of ethinyloestradiol and the progestogens norethisterone acetate and d-norgestrel. *Biochem Soc Trans.* Oct 1980;8(5):607-608.

15. Holschneider DP, Kumazawa T, Chen K, Shih JC. Tissue-specific effects of estrogen on monoamine oxidase A and B in the rat. *Life sciences.* 1998;63(3):155-160.

16. Gundlah C, Lu NZ, Bethea CL. Ovarian steroid regulation of monoamine oxidase-A and -B mRNAs in the macaque dorsal raphe and hypothalamic nuclei. *Psychopharmacology.* Mar 2002;160(3):271-282.

17. Smith LJ, Henderson JA, Abell CW, Bethea CL. Effects of ovarian steroids and raloxifene on proteins that synthesize, transport, and degrade serotonin in the raphe region of macaques. *Neuropsychopharmacology.* Nov 2004;29(11):2035-2045.

18. Ma ZQ, Bondiolotti GP, Olasmaa M, et al. Estrogen modulation of catecholamine synthesis and monoamine oxidase A activity in the human neuroblastoma cell line SK-ER3. *J Steroid Biochem Mol Biol.* Dec 1993;47(1-6):207-211.

19. Ma ZQ, Violani E, Villa F, Picotti GB, Maggi A. Estrogenic control of monoamine oxidase A activity in human neuroblastoma cells expressing physiological concentrations of estrogen receptor. *European journal of pharmacology.* Sep 15 1995;284(1-2):171-176.
20. Youdim MB, Bakhle YS. Monoamine oxidase: isoforms and inhibitors in Parkinson's disease and depressive illness. *Br. J. Pharmacol.* Jan 2006;147 Suppl 1:S287-296.
21. Meyer JH, Ginovart N, Boovariwala A, et al. Elevated monoamine oxidase a levels in the brain: an explanation for the monoamine imbalance of major depression. *Archives of general psychiatry.* Nov 2006;63(11):1209-1216.
22. Meyer JH, Wilson AA, Sagrati S, et al. Brain monoamine oxidase A binding in major depressive disorder: relationship to selective serotonin reuptake inhibitor treatment, recovery, and recurrence. *Arch Gen Psychiatry.* Dec 2009;66(12):1304-1312.
23. Johnson S, Stockmeier CA, Meyer JH, et al. The reduction of R1, a novel repressor protein for monoamine oxidase A, in major depressive disorder. *Neuropsychopharmacology.* 2011;36(10):2139-2148.
24. Freis ED. Mental depression in hypertensive patients treated for long periods with large doses of reserpine. *The New England journal of medicine.* Dec 16 1954;251(25):1006-1008.
25. Young SN, Smith SE, Pihl RO, Ervin FR. Tryptophan depletion causes a rapid lowering of mood in normal males. *Psychopharmacology (Berl).* 1985;87(2):173-177.
26. Bacher I, Houle S, Xu X, et al. Monoamine oxidase A binding in the prefrontal and anterior cingulate cortices during acute withdrawal from heavy cigarette smoking. *Arch Gen Psychiatry.* Aug 2011;68(8):817-826.
27. Sacher J, Wilson AA, Houle S, et al. Elevated brain monoamine oxidase A binding in the early postpartum period. *Arch. Gen. Psychiatry.* 2010;67(5):468-474.
28. Sacher J, Wilson AA, Rusjan PM, et al. Monoamine Oxidase A (MAO-A) Binding in Prefrontal and Anterior Cingulate Cortex in Postpartum Depression *Biol. Psychiatry.* 2011;69 (9):157S-157S.
29. Andres-Lacueva C, Shukitt-Hale B, Galli RL, Jauregui O, Lamuela-Raventos RM, Joseph JA. Anthocyanins in aged blueberry-fed rats are found centrally and may enhance memory. *Nutr Neurosci.* Apr 2005;8(2):111-120.
30. Joseph JA, Denisova NA, Arendash G, et al. Blueberry supplementation enhances signaling and prevents behavioral deficits in an Alzheimer disease model. *Nutr Neurosci.* Jun 2003;6(3):153-162.
31. Joseph JA, Shukitt-Hale B, Denisova NA, et al. Reversals of age-related declines in neuronal signal transduction, cognitive, and motor behavioral deficits with blueberry, spinach, or strawberry dietary supplementation. *J Neurosci.* Sep 15 1999;19(18):8114-8121.
32. Joseph JA, Shukitt-Hale B, Denisova NA, et al. Long-term dietary strawberry, spinach, or vitamin E supplementation retards the onset of age-related neuronal signal-transduction and cognitive behavioral deficits. *J Neurosci.* Oct 1 1998;18(19):8047-8055.
33. Kalt W, Blumberg JB, McDonald JE, et al. Identification of anthocyanins in the liver, eye, and brain of blueberry-fed pigs. *J. Agric. Food Chem.* Feb 13 2008;56(3):705-712.
34. Dowlati Y, Ravindran AV, Maheux M, Steiner M, Stewart DE, Meyer JH. No effect of oral tyrosine on total tyrosine levels in breast milk: implications for dietary supplementation in early postpartum. *Arch Womens Ment Health.* Dec 2014;17(6):541-548.
35. Dowlati Y, Ravindran AV, Maheux M, Steiner M, Stewart DE, Meyer JH. No effect of oral l-tryptophan or alpha-lactalbumin on total tryptophan levels in breast milk. *Eur. Neuropsychopharmacol.* Jun 2015;25(6):779-787.

36. Dowlati Y, Segal ZV, Ravindran AV, Steiner M, Stewart DE, Meyer JH. Effect of dysfunctional attitudes and postpartum state on vulnerability to depressed mood. *J. Affect. Disord.* Jun 2014;161:16-20.

37. Velten E, Jr. A laboratory task for induction of mood states. *Behav. Res. Ther.* Nov 1968;6(4):473-482.

38. Clark DM. The Velten Mood Induction Procedure and cognitive models of depression: a reply to Riskind and Rholes (1985). *Behav. Res. Ther.* 1985;23(6):667-669.

39. Association AP. *Diagnostic and Statistical Manual of Mental Disorders.* Vol Fourth Edition. Washington, DC: American Psychiatric Association; 1994.

40. Adewuya AO. Early postpartum mood as a risk factor for postnatal depression in Nigerian women. *Am J Psychiatry.* Aug 2006;163(8):1435-1437.

41. Hannah P, Adams D, Lee A, Glover V, Sandler M. Links between early post-partum mood and post-natal depression. *Br. J. Psychiatry.* Jun 1992;160:777-780.

42. Brockington IF, Cernik KF, Schofield EM, Downing AR, Francis AF, Keelan C. Puerperal Psychosis. Phenomena and diagnosis. *Arch Gen Psychiatry.* Jul 1981;38(7):829-833.

43. Kendell RE, Chalmers JC, Platz C. Epidemiology of puerperal psychoses. *Br J Psychiatry.* May 1987;150:662-673.

44. O'Hara MW, Swain A. Rates and risk of postpartum depression - a meta analysis. *International Review of Psychiatry.* 1996;8:37-54.

45. Antenatal and postnatal mental health: Clinical management and service guidance. In: Excellence NIfHaC, edNICE clinical guideline 45. London2007:4.

46. Pop VJ, Essed GG, de Geus CA, van Son MM, Komproe IH. Prevalence of post partum depression--or is it post-puerperium depression? *Acta Obstet Gynecol Scand.* Jul 1993;72(5):354-358.

47. Carothers AD, Murray L. Estimating psychiatric morbidity by logistic regression: application to post-natal depression in a community sample. *Psychol Med.* Aug 1990;20(3):695-702.

48. Kornstein SG. The evaluation and management of depression in women across the life span. *J Clin Psychiatry.* 2001;62 Suppl 24:11-17.

49. World_Health_Organization. *The Global Burden of Disease: 2004 Update.* Switzerland: Department of Health Statistics and Informatics, Information Evidence and Research Cluster, WHO;2008.

50. Comtois KA, Schiff MA, Grossman DC. Psychiatric risk factors associated with postpartum suicide attempt in Washington State, 1992-2001. *Am J Obstet Gynecol.* Aug 2008;199(2):120 e121-125.

51. Kumar R, Robson KM. A prospective study of emotional disorders in childbearing women. *Br J Psychiatry.* Jan 1984;144:35-47.

52. Philipps LH, O'Hara MW. Prospective study of postpartum depression: 4 1/2-year follow-up of women and children. *J Abnorm Psychol.* May 1991;100(2):151-155.

53. Nott PN. Extent, timing and persistence of emotional disorders following childbirth. *Br J Psychiatry.* Oct 1987;151:523-527.

54. Warner R, Appleby L, Whitton A, Faragher B. Demographic and obstetric risk factors for postnatal psychiatric morbidity. *Br J Psychiatry.* May 1996;168(5):607-611.

55. Horowitz JA, Goodman J. A longitudinal study of maternal postpartum depression symptoms. *Res Theory Nurs Pract.* Summer-Fall 2004;18(2-3):149-163.

56. Goodman JH. Postpartum depression beyond the early postpartum period. *J Obstet Gynecol Neonatal Nurs.* Jul-Aug 2004;33(4):410-420.

57. Clatworthy J. The effectiveness of antenatal interventions to prevent postnatal depression in high-risk women. *J Affect Disord.* Mar;137(1-3):25-34.

58. Weissman AM, Levy BT, Hartz AJ, et al. Pooled analysis of antidepressant levels in lactating mothers, breast milk, and nursing infants. *Am J Psychiatry.* Jun 2004;161(6):1066-1078.

59. Turner KM, Sharp D, Folkes L, Chew-Graham C. Women's views and experiences of antidepressants as a treatment for postnatal depression: a qualitative study. *Fam. Pract.* Dec 2008;25(6):450-455.

60. Dennis CL, Hodnett E, Kenton L, et al. Effect of peer support on prevention of postnatal depression among high risk women: multisite randomised controlled trial. *BMJ.* 2009;338:a3064.

61. Astbury J, Brown S, Lumley J, Small R. Birth events, birth experiences and social differences in postnatal depression. *Australian journal of public health.* Jun 1994;18(2):176-184.

62. Adewuya AO, Fatoye FO, Ola BA, Ijaodola OR, Ibigbami SM. Sociodemographic and obstetric risk factors for postpartum depressive symptoms in Nigerian women. *Journal of psychiatric practice.* Sep 2005;11(5):353-358.

63. Saura J, Bleuel Z, Ulrich J, et al. Molecular neuroanatomy of human monoamine oxidases A and B revealed by quantitative enzyme radioautography and in situ hybridization histochemistry. *Neuroscience.* 1996;70(3):755-774.

64. Youdim MB, Edmondson D, Tipton KF. The therapeutic potential of monoamine oxidase inhibitors. *Nature reviews.* Apr 2006;7(4):295-309.

65. Westlund KN, Krakower TJ, Kwan SW, Abell CW. Intracellular distribution of monoamine oxidase A in selected regions of rat and monkey brain and spinal cord. *Brain Res.* May 28 1993;612(1-2):221-230.

66. Nelson DL, Herbet A, Glowinski J, Hamon M. [3H]Harmaline as a specific ligand of MAO A--II. Measurement of the turnover rates of MAO A during ontogenesis in the rat brain. *J Neurochem.* Jun 1979;32(6):1829-1836.

67. Nelson DL, Herbet A, Petillot Y, Pichat L, Glowinski J, Hamon M. [3H]Harmaline as a specific ligand of MAO A--I. Properties of the active site of MAO A from rat and bovine brains. *J Neurochem.* Jun 1979;32(6):1817-1827.

68. Saura J, Kettler R, Da Prada M, Richards JG. Quantitative enzyme radioautography with 3H-Ro 41-1049 and 3H-Ro 19- 6327 in vitro: localization and abundance of MAO-A and MAO-B in rat CNS, peripheral organs, and human brain. *J Neurosci.* 1992;12(5):1977-1999.

69. Fowler C, Oreland L. Substrate-Selective Interaction Between Monoamine Oxidase and Oxygen. In: Singer T, Von Korff R, Murphy D, eds. *Monoamine Oxidase: Structure, Function, and Altered Functions*. New York: Academic Press, Inc.; 1979:145-151.

70. Kinemuchi H, Fowler C, Tipton K. Substrate Specificities of the Two Forms of Monoamine Oxidase. In: Tipton K, Dostert P, Strolin-Benedetti M, eds. *Monoamine Oxidase and Disease: Prospects for Therapy with Reversible Inhibitors*. New York: Academic Press, Inc.; 1984:53-62.

71. Schoepp DD, Azzaro AJ. Specificity of endogenous substrates for types A and B monoamine oxidase in rat striatum. *J Neurochem.* Jun 1981;36(6):2025-2031.

72. White H, Tansik R. Characterization of Multiple Substrate Binding Sites of MAO. *Monoamine Oxidase: Structure, Function and Altered Functions*. New York: Academic Press, Inc.; 1979:129-144.

73. Konradi C, Svoma E, Jellinger K, Riederer P, Denney R, Thibault J. Topographic immunocytochemical mapping of monoamine oxidase-A, monoamine oxidase-B and tyrosine hydroxylase in human post mortem brain stem. *Neuroscience.* Sep 1988;26(3):791-802.

74. Luque JM, Kwan SW, Abell CW, Da Prada M, Richards JG. Cellular expression of mRNAs encoding monoamine oxidases A and B in the rat central nervous system. *J Comp Neurol.* 1995;363(4):665-680.

75. Fagervall I, Ross SB. A and B forms of monoamine oxidase within the monoaminergic neurons of the rat brain. *J Neurochem.* Aug 1986;47(2):569-576.

76. Adell A, Biggs TA, Myers RD. Action of harman (1-methyl-beta-carboline) on the brain: body temperature and in vivo efflux of 5-HT from hippocampus of the rat. *Neuropharmacology.* 1996;35(8):1101-1107.

77. Bel N, Artigas F. In vivo evidence for the reversible action of the monoamine oxidase inhibitor brofaromine on 5-hydroxytryptamine release in rat brain. *Naunyn Schmiedebergs Arch Pharmacol.* May 1995;351(5):475-482.

78. Celada P, Artigas F. Monoamine oxidase inhibitors increase preferentially extracellular 5-hydroxytryptamine in the midbrain raphe nuclei. A brain microdialysis study in the awake rat. *Naunyn Schmiedebergs Arch Pharmacol.* Jun 1993;347(6):583-590.

79. Curet O, Damoiseau-Ovens G, Sauvage C, et al. Preclinical profile of befloxatone, a new reversible MAO-A inhibitor. *J Affect Disord.* Dec 1998;51(3):287-303.

80. Haefely W, Burkard WP, Cesura AM, et al. Biochemistry and pharmacology of moclobemide, a prototype RIMA. *Psychopharmacology (Berl).* 1992;106 Suppl:S6-14.

81. Evrard A, Malagie I, Laporte AM, et al. Altered regulation of the 5-HT system in the brain of MAO-A knock-out mice. *Eur J Neurosci.* Mar 2002;15(5):841-851.

82. Houslay MD, Tipton KF. A kinetic evaluation of monoamine oxidase activity in rat liver mitochondrial outer membranes. *Biochem J.* Jun 1974;139(3):645-652.

83. Konradi C, Kornhuber J, Froelich L, et al. Demonstration of monoamine oxidase-A and -B in the human brainstem by a histochemical technique. *Neuroscience.* 1989;33(2):383-400.

84. Fagervall I, Ross SB. A and B forms of monoamine oxidase within the monoaminergic neurons of the rat brain. *J Neurochem.* 1986;47(2):569-576.

85. Finberg JP, Pacak K, Goldstein DS, Kopin IJ. Modification of cerebral cortical noradrenaline release by chronic inhibition of MAO-A. *J Neural Transm Suppl.* 1994;41:123-125.

86. Finberg JP, Pacak K, Kopin IJ, Goldstein DS. Chronic inhibition of monoamine oxidase type A increases noradrenaline release in rat frontal cortex. *Naunyn Schmiedebergs Arch Pharmacol.* 1993;347(5):500-505.

87. Adachi YU, Watanabe K, Higuchi H, Satoh T, Vizi ES. Oxygen inhalation enhances striatal dopamine metabolism and monoamineoxidase enzyme inhibition prevents it: a microdialysis study. *Eur J Pharmacol.* Jun 22 2001;422(1-3):61-68.

88. Brannan T, Prikhojan A, Martinez-Tica J, Yahr MD. In vivo comparison of the effects of inhibition of MAO-A versus MAO-B on striatal L-DOPA and dopamine metabolism. *J Neural Transm Park Dis Dement Sect.* 1995;10(2-3):79-89.

89. Finberg JP, Wang J, Goldstein DS, Kopin IJ, Bankiewicz KS. Influence of selective inhibition of monoamine oxidase A or B on striatal metabolism of L-DOPA in hemiparkinsonian rats. *J Neurochem.* Sep 1995;65(3):1213-1220.

90. Segal DS, Kuczenski R, Okuda C. Clorgyline-induced increases in presynaptic DA: changes in the behavioral and neurochemical effects of amphetamine using in vivo microdialysis. *Pharmacol Biochem Behav.* Jul 1992;42(3):421-429.

91. Colzi A, d'Agostini F, Kettler R, Borroni E, Da Prada M. Effect of selective and reversible MAO inhibitors on dopamine outflow in rat striatum: a microdialysis study. *J Neural Transm Suppl.* 1990;32:79-84.

92. Colzi A, d'Agostini F, Cesura AM, Da Prada M. Brain microdialysis in rats: a technique to reveal competition in vivo between endogenous dopamine and moclobemide, a RIMA antidepressant. *Psychopharmacology (Berl).* 1992;106 Suppl:S17-20.

93. Butcher SP, Fairbrother IS, Kelly JS, Arbuthnott GW. Effects of selective monoamine oxidase inhibitors on the in vivo release and metabolism of dopamine in the rat striatum. *J Neurochem.* 1990;55(3):981-988.

94. Wayment HK, Schenk JO, Sorg BA. Characterization of extracellular dopamine clearance in the medial prefrontal cortex: role of monoamine uptake and monoamine oxidase inhibition. *J Neurosci.* Jan 1 2001;21(1):35-44.

95. Moll G, Moll R, Riederer P, Gsell W, Heinsen H, Denney RM. Immunofluorescence cytochemistry on thin frozen sections of human substantia nigra for staining of monoamine oxidase A and monoamine oxidase B: a pilot study. *J Neural Transm Suppl.* 1990;32:67-77.

96. Ginovart N, Meyer JH, Boovariwala A, et al. Positron emission tomography quantification of [11C]-harmine binding to monoamine oxidase-A in the human brain. *J Cereb Blood Flow Metab.* Mar 2006;26(3):330-344.

97. First M, Spitzer R, Williams J, Gibbon M. *Structured Clinical Interview for DSM-IV-Non-Patient Edition (SCID-NP, Version 1.0)*. Washington, D.C.: American Psychiatric Press; 1995.

98. Grote SS, Moses SG, Robins E, Hudgens RW, Croninger AB. A study of selected catecholamine metabolizing enzymes: a comparison of depressive suicides and alcoholic suicides with controls. *J Neurochem.* 1974;23(4):791-802.

99. Gottfries CG, Oreland L, Wiberg A, Winblad B. Lowered monoamine oxidase activity in brains from alcoholic suicides. *J Neurochem.* 1975;25(5):667-673.

100. Mann JJ, Stanley M. Postmortem monoamine oxidase enzyme kinetics in the frontal cortex of suicide victims and controls. *Acta Psychiatr Scand.* 1984;69(2):135-139.

101. Sherif F, Marcusson J, Oreland L. Brain gamma-aminobutyrate transaminase and monoamine oxidase activities in suicide victims. *Eur Arch Psychiatry Clin Neurosci.* 1991;241(3):139-144.

102. Ordway GA, Farley JT, Dilley GE, et al. Quantitative distribution of monoamine oxidase A in brainstem monoamine nuclei is normal in major depression. *Brain Res.* 1999;847(1):71-79.

103. Johnson S, Stockmeier CA, Meyer JH, et al. The Reduction of R1, a Novel Repressor Protein for Monoamine Oxidase A, in Major Depressive Disorder. *Neuropsychopharmacology.* Jun 8 2011.

104. Johnson S, Stockmeier CA, Meyer JH, et al. The reduction of R1, a novel repressor protein for monoamine oxidase a, in major depressive disorder. *Neuropsychopharmacology.* Sep;36(10):2139-2148.

105. Ou XM, Chen K, Shih JC. Glucocorticoid and androgen activation of monoamine oxidase A is regulated differently by R1 and Sp1. *J Biol Chem.* Jul 28 2006;281(30):21512-21525.

106. Ou XM, Chen K, Shih JC. Monoamine oxidase A and repressor R1 are involved in apoptotic signaling pathway. *Proceedings of the National Academy of Sciences of the United States of America.* Jul 18 2006;103(29):10923-10928.

107. Barton DA, Esler MD, Dawood T, et al. Elevated brain serotonin turnover in patients with depression: effect of genotype and therapy. *Archives of general psychiatry.* Jan 2008;65(1):38-46.

108. Saura J, Kettler R, Da Prada M, Richards JG. Quantitative enzyme radioautography with 3H-Ro 41-1049 and 3H-Ro 19-6327 in vitro: localization and abundance of MAO-A and MAO-B in rat CNS, peripheral organs, and human brain. *J Neurosci.* May 1992;12(5):1977-1999.

109. Hendrick V, Altshuler LL, Suri R. Hormonal changes in the postpartum and implications for postpartum depression. *Psychosomatics.* Mar-Apr 1998;39(2):93-101.

110. Bergstrom M, Westerberg G, Kihlberg T, Langstrom B. Synthesis of some 11C-labelled MAO-A inhibitors and their in vivo uptake kinetics in rhesus monkey brain. *Nuclear medicine and biology.* 1997;24(5):381-388.

111. Bergstrom M, Westerberg G, Langstrom B. 11C-harmine as a tracer for monoamine oxidase A (MAO-A): in vitro and in vivo studies. *Nuclear medicine and biology.* 1997;24(4):287-293.

112. Young SN, Smith SE, Pihl RO, Ervin FR. Tryptophan depletion causes a rapid lowering of mood in normal males. *Psychopharmacology.* 1985;87(2):173-177.

113. Delgado PL, Miller HL, Salomon RM, et al. Monoamines and the mechanism of antidepressant action: effects of catecholamine depletion on mood of patients treated with antidepressants. *Psychopharmacol Bull.* 1993;29(3):389-396.

114. Benkelfat C, Ellenbogen MA, Dean P, Palmour RM, Young SN. Mood-lowering effect of tryptophan depletion. Enhanced susceptibility in young men at genetic risk for major affective disorders. *Archives of general psychiatry.* 1994;51(9):687-697.

115. Oldman A, Walsh A, Salkovskis P, Laver D, Cowen P. Effect of acute tryptophan depletion on mood and appetite in healthy female volunteers. *J Psychopharmacol.* 1994;8(1):8-13.

116. Ellenbogen MA, Young SN, Dean P, Palmour RM, Benkelfat C. Mood response to acute tryptophan depletion in healthy volunteers: sex differences and temporal stability. *Neuropsychopharmacology.* 1996;15(5):465-474.

117. Laruelle M, D'Souza CD, Baldwin RM, et al. Imaging D2 receptor occupancy by endogenous dopamine in humans. *Neuropsychopharmacology.* 1997;17(3):162-174.

118. Verhoeff NP, Kapur S, Hussey D, et al. A simple method to measure baseline occupancy of neostriatal dopamine d(2) receptors by dopamine in vivo in healthy subjects. *Neuropsychopharmacology.* 2001;25(2):213-223.

119. Mendels J, Frazer A. Brain biogenic amine depletion and mood. *Archives of general psychiatry.* Apr 1974;30(4):447-451.

120. Burrell RH. Depression associated with reserpine therapy. *The New Zealand medical journal.* Jun 1956;55(307):228-231.

121. Gawryluk JW, Wang JF, Andreazza AC, Shao L, Young LT. Decreased levels of glutathione, the major brain antioxidant, in post-mortem prefrontal cortex from patients with psychiatric disorders. *Int J Neuropsychopharmacol.* Feb 2011;14(1):123-130.

122. Andreazza AC, Shao L, Wang JF, Young LT. Mitochondrial complex I activity and oxidative damage to mitochondrial proteins in the prefrontal cortex of patients with bipolar disorder. *Arch. Gen. Psychiatry.* Apr 2010;67(4):360-368.

123. Young SN, Leyton M. The role of serotonin in human mood and social interaction. Insight from altered tryptophan levels. *Pharmacol Biochem Behav.* Apr 2002;71(4):857-865.

124. Leyton M, Young SN, Pihl RO, et al. Effects on mood of acute phenylalanine/tyrosine depletion in healthy women. *Neuropsychopharmacology.* 2000;22(1):52-63.

125. Hawkins RA, O'Kane RL, Simpson IA, Vina JR. Structure of the blood-brain barrier and its role in the transport of amino acids. *J Nutr.* Jan 2006;136(1 Suppl):218S-226S.

126. Fernstrom JD. Dietary precursors and brain neurotransmitter formation. *Annu. Rev. Med.* 1981;32:413-425.

127. Cooper J, Bloom F, Roth R. *The Biochemical Basis of Neuropharmacology*. 6 ed. New York: Oxford University Press; 1991.

128. Halvorsen B, Carlsen M, Phillips K, et al. Content of redox-active compounds (ie, antioxidants) in foods consumed in the United States. *Am J Clin Nutr.* 2006;84:95-135.

129. Wu X, Beecher G, Holden J, Haytowitz D, Gebhardt S, Prior R. Concentrations of Anthocyanins in Common Foods in the United States and Estimation of Normal Consumuption. *J Agric Food Chem.* 2006;54:4069-4075.

130. Kalt W, Blumberg J, McDonald J, et al. Identification of Anthocyanins in the Liver, Eye and Brain of Blueberry-Fed Pigs. *J Agric Food Chem.* 2008;56:705-712.

131. Lemons JA, Reyman D, Moye L. Amino acid composition of preterm and term breast milk during early lactation. *Early Hum Dev.* Oct 1983;8(3-4):323-329.

132. Macy IG. Composition of human colostrum and milk. *Am. J. Dis. Child.* Oct 1949;78(4):589-603.

133. Wurtman JJ, Fernstrom JD. Free amino acid, protein, and fat contents of breast milk from Guatemalan mothers consuming a corn-based diet. *Early Hum. Dev.* Mar 1979;3(1):67-77.

134. Svanberg U, Gebre-Medhin M, Ljungqvist B, Olsson M. Breast milk composition in Ethiopian and Swedish mothers. III. Amino acids and other nitrogenous substances. *Am J Clin Nutr.* Apr 1977;30(4):499-507.

135. Ingram RE, Ritter J. Vulnerability to depression: cognitive reactivity and parental bonding in high-risk individuals. *J. Abnorm. Psychol.* Nov 2000;109(4):588-596.

136. Gilboa-Schechtman E, Revelle W, Gotlib IH. Stroop interference following mood induction: Emotionality, mood congruence and concern relevance. *Cognitive Therapy and Research.* 2000;24:491-502.

137. Gomez R, Cooper A, Gomez A. Susceptibility to positive and negative mood states: Test of Eysenck's, Gray's and Newman's theories. *Personality and Individual Differences.* 2000;29:351-366.

138. Segal ZV, Gemar M, Williams S. Differential cognitive response to a mood challenge following successful cognitive therapy or pharmacotherapy for unipolar depression. *J. Abnorm. Psychol.* Feb 1999;108(1):3-10.

139. Segal ZV, Kennedy S, Gemar M, Hood K, Pedersen R, Buis T. Cognitive reactivity to sad mood provocation and the prediction of depressive relapse. *Arch Gen Psychiatry.* Jul 2006;63(7):749-755.

140. Banderet LE, Lieberman HR. Treatment with tyrosine, a neurotransmitter precursor, reduces environmental stress in humans. *Brain research bulletin.* Apr 1989;22(4):759-762.

141. Deijen JB, Orlebeke JF. Effect of tyrosine on cognitive function and blood pressure under stress. *Brain research bulletin.* 1994;33(3):319-323.

142. Magill RA, Waters WF, Bray GA, et al. Effects of tyrosine, phentermine, caffeine D-amphetamine, and placebo on cognitive and motor performance deficits during sleep deprivation. *Nutritional neuroscience.* Aug 2003;6(4):237-246.

143. Owasoyo JO, Neri DF, Lamberth JG. Tyrosine and its potential use as a countermeasure to performance decrement in military sustained operations. *Aviation, space, and environmental medicine.* May 1992;63(5):364-369.

144. Shurtleff D, Thomas JR, Schrot J, Kowalski K, Harford R. Tyrosine reverses a cold-induced working memory deficit in humans. *Pharmacology, biochemistry, and behavior.* Apr 1994;47(4):935-941.

145. Sutton EE, Coill MR, Deuster PA. Ingestion of tyrosine: effects on endurance, muscle strength, and anaerobic performance. *International journal of sport nutrition and exercise metabolism.* Apr 2005;15(2):173-185.

146. Mahoney CR, Castellani J, Kramer FM, Young A, Lieberman HR. Tyrosine supplementation mitigates working memory decrements during cold exposure. *Physiology & behavior.* Nov 23 2007;92(4):575-582.

147. O'Brien C, Mahoney C, Tharion WJ, Sils IV, Castellani JW. Dietary tyrosine benefits cognitive and psychomotor performance during body cooling. *Physiology & behavior.* Feb 28 2007;90(2-3):301-307.

148. Markus CR, Firk C, Gerhardt C, Kloek J, Smolders GF. Effect of different tryptophan sources on amino acids availability to the brain and mood in healthy volunteers. *Psychopharmacology (Berl).* Nov 2008;201(1):107-114.

149. Adewuya AO. Early postpartum mood as a risk factor for postnatal depression in Nigerian women. *Am. J. Psychiatry.* Aug 2006;163(8):1435-1437.

150. American Psychiatric Association, Diagnostic and statistical manual of mental disorders, Fifth Edition (DSM-V)Washington, DC: American Psychiatric Association; 2013.

151. Beck AT, Ward CH, Mendelson M, Mock J, Erbaugh J. An inventory for measuring depression. *Arch. Gen. Psychiatry.* Jun 1961;4:561-571.

152. Weissman AN. The Dysfunctional Attitude Scale:a validation study. *Diss. Abstr. Int.* 1979;40:1389B-1390B.

153. Weissman AN, Beck AT. Development and validation of the dysfunctional attitude scale: a preliminary investigation. *Annual meeting of the American Educational Research Association.* Vol Toronto, Ontario, Canada1978.

154. Costa PT, Jr., McCrae RR. Stability and change in personality assessment: the revised NEO Personality Inventory in the year 2000. *J. Pers. Assess.* Feb 1997;68(1):86-94.

155. Hamilton M. A rating scale for depression. *J. Neurol. Neurosurg. Psychiatry.* Feb 1960;23:56-62.

156. Cox JL, Holden JM, Sagovsky R. Detection of postnatal depression. Development of the 10-item Edinburgh Postnatal Depression Scale. *Br. J. Psychiatry.* 1987;150:782-786.

157. Devilly GJ, Borkovec TD. Psychometric properties of the credibility/expectancy questionnaire. *J. Behav. Ther. Exp. Psychiatry.* Jun 2000;31(2):73-86.

158. Buysse DJ, Reynolds CF, 3rd, Monk TH, Berman SR, Kupfer DJ. The Pittsburgh Sleep Quality Index: a new instrument for psychiatric practice and research. *Psychiatry Res.* May 1989;28(2):193-213.

159. Kendell RE, McGuire RJ, Connor Y, Cox JL. Mood changes in the first three weeks after childbirth. *J. Affect. Disord.* Dec 1981;3(4):317-326.

160. McNair DM, Lorr M, Droppleman LF. *EITS Manual for the Profile of Mood States*. San Diego: Educational and Industrial Testing Service; 1971.

161. Spielberger CD, Gorsuch RL, Lushene RE. *Manual for the State-Trait Anxiety Inventory*. Palo Alto, CA Consulting Psychologists Press; 1970.

162. Frost RO, Green ML. VeltenMood Induction Procedure Effects: Duration and Postexperimental Removal. *Personality and Social Psychology Bulletin.* 1982;8:341-347.

163. Stein GS. The pattern of mental change and body weight change in the first post-partum week. *J. Psychosom. Res.* 1980;24(3-4):165-171.

164. Kennerley H, Gath D. Maternity blues. I. Detection and measurement by questionnaire. *Br. J. Psychiatry.* Sep 1989;155:356-362.

165. Radloff LS. The CES-D scale, a self-report depression scale for research in the general population. *J Appl Psychol Measurement.* 1977;1:385-401.
